# Murine nuclear tyrosyl-tRNA synthetase deficiency leads to fat storage deficiency and hearing loss

**DOI:** 10.1016/j.jbc.2024.107756

**Published:** 2024-09-12

**Authors:** Julia A. Jones, Jiadong Zhou, Jianjie Dong, Salvador Huitron-Resendiz, Ely Boussaty, Eduardo Chavez, Na Wei, Calin Dan Dumitru, Yosuke Morodomi, Taisuke Kanaji, Allen F. Ryan, Rick Friedman, Tong Zhou, Sachiko Kanaji, Matthew Wortham, Simon Schenk, Amanda J. Roberts, Xiang-Lei Yang

**Affiliations:** 1 Department of Molecular Medicine, The Scripps Research Institute, La Jolla, California, USA; 2 Animal Models Core Facility, The Scripps Research Institute, La Jolla, California, USA; 3 Department of Otolaryngology - Head and Neck Surgery, University of California San Diego, La Jolla, California, USA; 4 Department of Physiology and Cell Biology, University of Nevada, Reno School of Medicine, Reno, Nevada, USA; 5 Department of Pediatrics, Pediatric Diabetes Research Center, University of California San Diego, La Jolla, California, USA; 6 Department of Orthopaedic Surgery, University of California San Diego, La Jolla, California, USA

**Keywords:** aminoacyl tRNA synthetase, adipose tissue, metabolism, insulin, hearing

## Abstract

Aminoacyl-tRNA synthetases are fundamental to the translation machinery with emerging roles in transcriptional regulation. Previous cellular studies have demonstrated tyrosyl-tRNA synthetase (*YARS1* or TyrRS) as a stress response protein through its cytosol-nucleus translocation to maintain cellular homeostasis. Here, we established a mouse model with a disrupted TyrRS nuclear localization signal, revealing its systemic impact on metabolism. Nuclear TyrRS deficiency (*Yars*^ΔNLS^) led to reduced lean mass, reflecting a mild developmental defect, and reduced fat mass, possibly due to increased energy expenditure. Consistently, *Yars*^ΔNLS^ mice exhibit improved insulin sensitivity and reduced insulin levels, yet maintain normoglycemia, indicative of enhanced insulin action. Notably, *Yars*^ΔNLS^ mice also develop progressive hearing loss. These findings underscore the crucial function of nuclear TyrRS in the maintenance of fat storage and hearing and suggest that aminoacyl-tRNA synthetases' regulatory roles can affect metabolic pathways and tissue-specific health. This work broadens our understanding of how protein synthesis interconnects metabolic regulation to ensure energy efficiency.

Aminoacyl-tRNA synthetases (aaRSs) are a critical enzyme family for protein synthesis, a fundamental process of metabolism. They are responsible for "charging" tRNA molecules with the correct amino acids; the charged tRNAs are then used for translating the genetic code into proteins (1). Beyond their central role in protein synthesis, during evolution, many aaRSs have acquired additional regulatory functions acting through signaling (2, 3), gene expression regulation (4, 5), and response to cellular stress (6, 7) to influence cellular metabolism.

Various regulatory functions have been reported for tyrosyl-tRNA synthetase (TyrRS) (8, 9), including stress response acting as a transcription modulator in the nucleus (10, 11, 12, 13). A nuclear localization signal (NLS) sequence, conserved from insects to humans, is found in TyrRS (14). The NLS is in the anticodon recognition domain of TyrRS and overlaps with a tRNA-binding site (Fig. 1*A*). Under stress conditions where tRNA are depleted due to cleavage or retrograde transport (15, 16, 17), TyrRS becomes unbound with tRNA, and the NLS is exposed for recognition by importins. Thus, the location of the NLS appears strategic in allowing TyrRS to sense and respond to stress conditions through nuclear translocation (12, 14). Nuclear TyrRS has been shown to regulate transcription to restore cellular homeostasis under oxidative stress conditions by upregulating DNA damage response genes and downregulating translation-related genes (10, 11, 12). Interestingly, oxidative stress is associated with the development and progression of metabolic diseases (18, 19, 20). Reactive oxygen species (ROS) are important for cell signaling, but their accumulation can lead to DNA damage, lipid peroxidation, protein damage, and overall redox imbalance, which have been associated with obesity and many other metabolic diseases and even hearing loss (20, 21). In cells, TyrRS translocation can also be stimulated by a variety of stress conditions beyond oxidative stress, including ER stress and nutrient deprivation (12, 13, 14, 22). However, how this translates to the diverse physiological stressors in a mammalian animal model remains unexplored.

**Figure 1:**
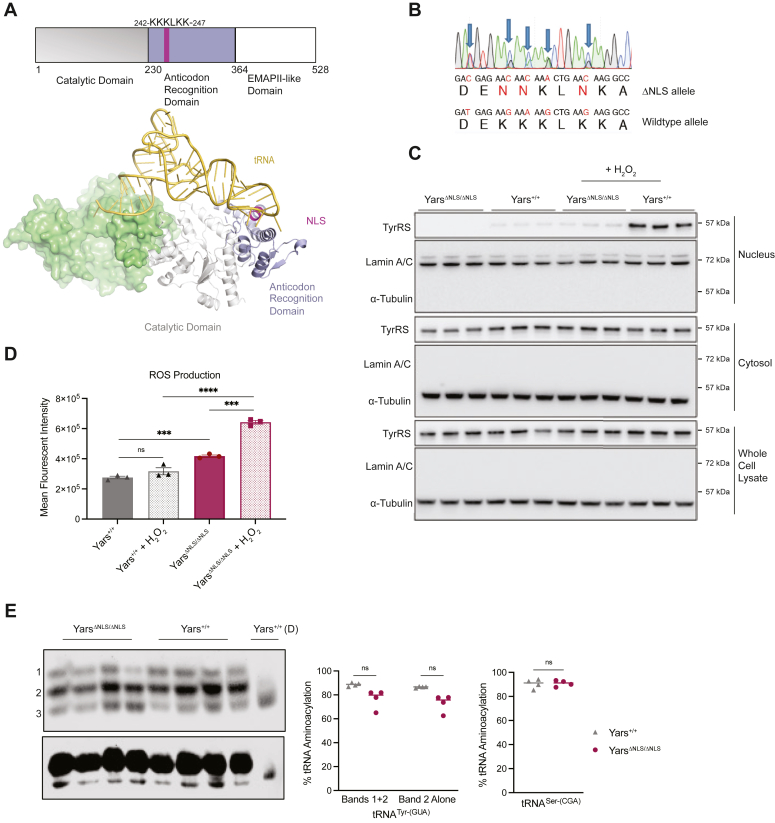
**Generation of a nuclear TyrRS-deficient mouse model.***A*, TyrRS domain architecture highlighting the NLS location in the anticodon recognition domain. The dimer structure of TyrRS with tRNA^Tyr^ modeled based on PDB:1N3L and 1J1U without the EMAP-II domain. *B*, RT-PCR mRNA sequencing confirmation of the NLS mutation from a heterozygous, *Yars*^*ΔNLS/+*^*,* mouse. *C*, Western blot of primary homozygous, *Yars*^*ΔNLS/ΔNLS*^, and WT, *Yars*^*+/+*^, MEFs cell fractionated with or without 250 μM H_2_O_2_ treatment. Lamin A/C: nuclear marker, α-Tubulin: cytosolic marker. *D*, ROS production measured by CM-H_2_DCFDA dye with or without 10 μM H_2_O_2_ treatment for 30 min. Two-way ANOVA with Fisher’s LSD test. *E*, acidic northern blot separating acylated and deacylated tRNA^Tyr-(GUA)^ in MEFs and quantification of the charged species (Band 1 and 2) and major uncharged species (Band 3). Blot reprobed for tRNA^Ser-(CGA)^. (*D*): Deacetylated. Four independent embryos per genotype. Brown-Forsythe ANOVA using Dunnett’s T3 multiple comparisons and Unpaired Welch’s *t* test.

Intriguingly, biallelic recessive mutations in aaRSs often result in multisystem disorders that mimic features of many metabolic diseases (23, 24). Some aaRS mutations affect a variety of organs while others appear to be tissue-specific (24). Multiple biallelic mutations in TyrRS-encoding gene *YARS*1 lead to multisystem disorders exhibiting metabolic signatures like hypothyroidism, hypoglycemia, anemia, liver disease, and hearing impairments (25, 26, 27, 28, 29). Loss of enzymatic activity and stability are proposed explanations of these biallelic mutations but cannot fully explain the clinical heterogeneity and tissue specificity observed. Additionally, a few recent studies demonstrated some aaRS mutations do not affect the enzyme activity or global protein synthesis, opening the possibility that other regulatory functions of aaRS could play a role in these disorders (30, 31, 32). TyrRS is also one of the several aaRSs causatively linked to a hereditary neuropathy called Charcot-Marie-Tooth (CMT) disease through monoallelic dominant mutations (33). The implication of nuclear TyrRS in CMT arose from the observation that excluding mutant TyrRS from the nucleus prevented the hallmark phenotypes of CMT in a *Drosophila* model (34). However, the physiological role of nuclear TyrRS has not been investigated.

In this study, we established a mouse model to define the systemic impact of nuclear TyrRS and to understand its contribution to disease syndromes associated with its genetic mutations. We demonstrated that nuclear TyrRS-deficient mice have functional changes across different tissues, including hearing loss, enhanced energy expenditure, and reduced adiposity, impacting insulin signaling. Yet, the exact molecular mechanisms require further investigation. Overall, our study suggests that aaRS nuclear functions can be an important factor for energy homeostasis and hearing maintenance.

## Results

### *Yars*^ΔNLS^ MEFs exhibit metabolic dysregulation

To explore the role of nuclear TyrRS in a mammalian model, a nuclear deficient mouse model was generated by mutating the NLS from ^242^KKKLKK^247^ to ^242^NNKLNK^247^ (referred to as *Yars*^ΔNLS^) (Fig. S1*A*), which predominantly excludes TyrRS from the nucleus while allowing enzymatic activity *in vitro* (14). The expression of the mutant allele was demonstrated by RT-PCR and sequencing (Fig. 1*B*). Using cell fractionation followed by Western blot, we verified the nuclear import deficiency of TyrRS in *Yars*^ΔNLS^ mouse embryonic fibroblasts, MEFs (Fig. 1*C*). As established previously in other cell types, the nuclear import of TyrRS was stimulated upon hydrogen peroxide treatment in WT MEFs, but minimal levels of TyrRS were found in mutant *Yars*^ΔNLS^ MEFs with or without oxidative stress (Fig. 1*C*). Consistent with the role of nuclear TyrRS protecting cells against oxidative stress and overstimulation of protein synthesis (10, 11, 12), mutant *Yars*^ΔNLS^ MEFs exhibited increased ROS at basal and stressed conditions (Fig. 1*D*), as well as increased protein synthesis and enhanced mitochondrial respiration (Fig. S1, *B* and *C*). Thus, we confirmed metabolic dysregulation from disrupting nuclear TyrRS import in *Yars*^ΔNLS^ MEFs.

### *Yars*^ΔNLS^ results in a mild decrease in tRNA^Tyr^ charging level in MEFs and liver

The enzymatic activity of TyrRS in MEFs was evaluated using acidic gel northern blot that separates charged and uncharged tRNA during electrophoresis. The MEF samples exhibited two forms of charged tRNA^Tyr (GUA)^ (Fig. 1*E*), presumably corresponding to differences in the modification status of the tRNA. All tRNAs especially tRNA^Tyr^ are extensively modified, which could lead to different forms of tRNA^Tyr^ as seen in our MEF samples (35). The top and middle bands (band 1 and 2) correspond to a minor and a major form of the charged tRNA^Tyr^, respectively; the bottom band (band 3) corresponds to the major form of the uncharged tRNA^Tyr^ (Fig. 1*E*). It is possible that band 2 also contains a small amount of uncharged tRNA^Tyr^ in the minor form. Using two ways of analysis that considered both forms (band 1 + 2) or only the major form (band 2 only) of the charged tRNA^Tyr^, the results indicated that about 80 to 90% of tRNA^Tyr^ were charged in the WT MEFs and the charged level of tRNA^Tyr^ was slightly decreased in the *Yars*^ΔNLS^ cells (Fig. 1*E*). In contrast, no decrease was observed in the charging level of a noncognate tRNA in the mutant cells (Fig. 1*E*). Thus, this slight decrease is specific to the cognate tRNA and consistent with the initial characterization of this mutation that indicated a small impact on tRNA binding and charging (14). In contrast to MEF samples, only one band of the aminoacylated tRNA^Tyr (GUA)^ was observed in the liver tissue, where the level of tRNA^Tyr^ aminoacylation is also slightly decreased in *Yars*^ΔNLS^ compared with the WT animals (Fig. S1*D*). At birth, homozygous *Yars*^ΔNLS^ mice had a slightly smaller body weight (Fig. S1*E*) and at wean were at approximate Mendelian ratio (Fig. S1*F*), suggesting that the slight decrease in tRNA^Tyr^ aminoacylation does not significantly impact survival but may have some impact on development.

### *Yars*^ΔNLS^ mice are smaller

**Figure 2:**
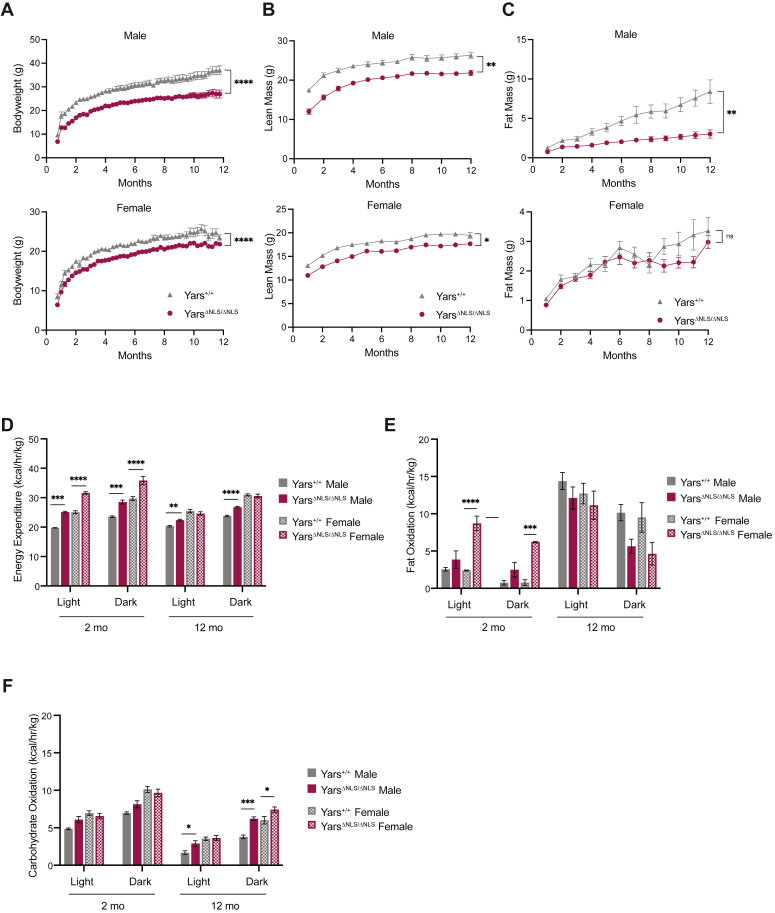
***Yars***^**ΔNLS**^**mice exhibit reduced body weight due to both lean and fat mass differences with an overall increased metabolic rate with varying substrate oxidation in an age-dependent manner.***A*, male and female body weights measured weekly for 47 weeks starting at 3 weeks of age. Mixed-effects 2-way ANOVA with Sidak’s analysis. *B*, lean mass of male and female mice monthly for 11 months measured by EchoMRI. Mixed-effects 2-way ANOVA with Sidak’s analysis. *C*, fat mass of male and female mice monthly for 11 months measured by EchoMRI. Mixed-effects 2-way ANOVA with Sidak’s analysis. For *A*–*C*, male: N = 8 for *Yars*^*+/+*^ and N = 13 for *Yars*^*ΔNLS/ΔNLS*^; female: N = 8 for *Yars*^*+/+*^ and *Yars*^*ΔNLS/ΔNLS*^. *D*, average energy expenditure (EE) calculated from gas exchange in the metabolic chambers (CLAMS) at 2 and 12 months for both sexes over the light and dark cycles. *E*, average fat oxidation rate calculated from EE and respiratory exchange ratio (RER) at 2 and 12 months for both sexes over the dark and light cycles. *F*, average carbohydrate oxidation rates calculated at 2 and 12 months for both sexes over the light and dark cycles. For *D*–*F*, statistical differences were calculated with a two-way ANOVA with Tukey comparisons analysis. Two-month sample size: male: *Yars*^*+/+*^ N = 8, *Yars*^*ΔNLS/ΔNLS*^ N = 13; female: N = 7 for *Yars*^*+/+*^ and *Yars*^*ΔNLS/ΔNLS*^; Twelve-month sample size: Male and female: N = 4 for *Yars*^*+/+*^ and *Yars*^*ΔNLS/ΔNLS*^. Time in chamber: 72 h for 2-month-old mice; 144 h for 12-month-old.

Throughout the homozygous *Yars*^ΔNLS^ mice lifetime, they have a smaller body weight in both sexes (Fig. 2*A*). The heterozygous mice also exhibit a smaller body weight to a lesser degree (data not shown); therefore, we focused on the homozygous mice. The body composition suggested this body weight difference was attributed to a constant lean mass difference and a shorter body length (Figs. 2*B* and S2*A*), indicating the *Yars*^ΔNLS^ mouse was overall smaller. Yet, no gross lesions were found in the organs examined during a necropsy analysis at 2 months. As typically seen in zebrafish and mouse models, homozygous aaRS knockouts are nonviable and the knockdowns exhibit developmental defects (36, 37, 38, 39), http://www.informatics.jax.org. However, in those knockdown or knockout studies, charged tRNA levels are presumably reduced more than what we observed in *Yars*^ΔNLS^ samples (∼10%); therefore, although the slight reduction in tRNA charging observed in the *Yars*^ΔNLS^ mice (Figs. 1*E* and S1*D*) may contribute to the smaller stature, there may be other factors beyond tRNA charging impacting the body weight.

### *Yars*^ΔNLS^ mice unable to accumulate fat likely due to enhanced fuel utilization

Curiously, during adulthood in *Yars*^ΔNLS^ males, the mice exhibit an inability to accumulate adipose tissue, as evidenced by the fact that their fat mass does not increase (Fig. 2*C*). In *Yars*^ΔNLS^ females, the trend is not as obvious (Fig. 2*C*); however, when the female mice were put on an increased caloric diet for 12 weeks, there was a significant difference in fat mass resembling the male mice on a chow diet (Fig. S2*B*). The body weight differences cannot be attributed to reduced food consumption or heightened physical activity. In fact, *Yars*^ΔNLS^ mice have a tendency for increased food intake and decreased physical activity as they age (Table 1). To assess the apparent energy deficit, we utilized the Comprehensive Lab Animal Monitoring System (CLAMS) at 2 and 12 months of age. At 2 months, there was an overall increase in energy expenditure indicating an increase in metabolic demands in both *Yars*^ΔNLS^ mice sexes (Fig. 2*D*). The respiratory exchange ratio (RER) at 2 months showed no obvious difference in males, but female RER decreased hinting at more fat utilization for the mutants (Fig. S2*C*). To further dissect the primary fuel selection, fat and carbohydrate oxidation rates were calculated. Consistent with RER, *Yars*^ΔNLS^ females exhibited a substantially enhanced fat oxidation at 2 months (Fig. 2*E*). The increase in energy expenditure was maintained at 12 months in males to a lesser extent (Fig. 2*D*). The RER was also higher, indicating higher carbohydrate utilization for the mutant mice at 12 months (Fig. S2*C*). Consistently, the mutants exhibited enhanced carbohydrate oxidation (Fig. 2*F*), which corresponded to less adipose availability in *Yars*^ΔNLS^ mice at 12 months (Fig. 2*C*). Regardless of the less dramatic adipose difference in females, overall, the *Yars*^ΔNLS^ mice display a similar metabolic profile across both sexes. This points to metabolic alterations in *Yars*^ΔNLS^ mice where the smaller body weight correlates with an increased energy demand and fuel utilization, suggesting a role of nuclear TyrRS in metabolic homeostasis.

**Table 1:** Average activity (beam breaks), food intake (grams), and water intake (milliliters) at 2 and 12 months

Sex	Genotype	Average activity (2 months)	Average activity (12 months)	Food intake (2 months)	Food intake (12 months)	Water intake (2 months)	Water intake (12 months)
Male	Yars^+/+^	32,474.44 ± 3012.18	20,813.81 ± 2661.16	11.15 ± 0.39	19.75 ± 0.22	8.02 ± 0.76	11.85 ± 0.14
	Yars^ΔNLS/ΔNLS^	25,493 ± 1623.54	16,166.17 ± 2168.18	11.1 ± 0.58	23.77 ± 0.39	8.06 ± 0.58	15 ± 0.20
	Significance	ns	ns	ns	∗∗∗	ns	∗∗∗∗
Female	Yars^+/+^	33,134.26 ± 3380.46	33,795.79 ± 4766.97	12.11 ± 0.58	22.64 ± 0.30	8.51 ± 1.14	16.79 ± 0.11
	Yars^ΔNLS/ΔNLS^	31,763.5 ± 2152.39	24,392.96 ± 2536.89	10.89 ± 0.78	24.36 ± 0.35	6.34 ± 0.64	19 ± 0.16
	Significance	ns	ns	ns	∗∗	ns	∗∗∗∗

Mice in chamber for 72 h at 2 months and 144 h at 12 months. ± = sem; (∗∗∗∗) *p* < 0.0001, (∗∗∗) *p* < 0.001, (∗∗) *p* < 0.01, (∗) *p* < 0.05, (ns) not significant; Unpaired Welch's *t* test.

We also investigated additional lipid properties that might contribute or be associated with the reduced adiposity in the *Yars*^ΔNLS^ mice, such as an accumulation of lipids in circulation or increased lipid distribution in other tissues. Serum cholesterol, triglycerides, and free fatty acids were not elevated (Fig. S2*D*), and there was not an overt lipid accumulation in liver by H&E staining to support the notion of enhanced lipolysis or abnormal lipid deposition (Fig. S2*E*). Epididymal adipocyte cell size was similar implying adipose differentiation was unaltered (Fig. S2*F*). Consistently, *ex vivo* differentiation of primary MEFs into adipocytes showed no difference in adipogenesis capacity (Fig. S2*G*). In addition, brown adipose, skeletal muscle, and liver masses were not disproportionately sized in *Yars*^ΔNLS^ mice (Fig. S2*H*). Taken together, despite the reduced adiposity, *Yars*^ΔNLS^ mice exhibit normal lipid distribution and adipogenesis.

### *Yars*^ΔNLS^ mice display enhanced insulin sensitivity and reduced circulating insulin levels

**Figure 3:**
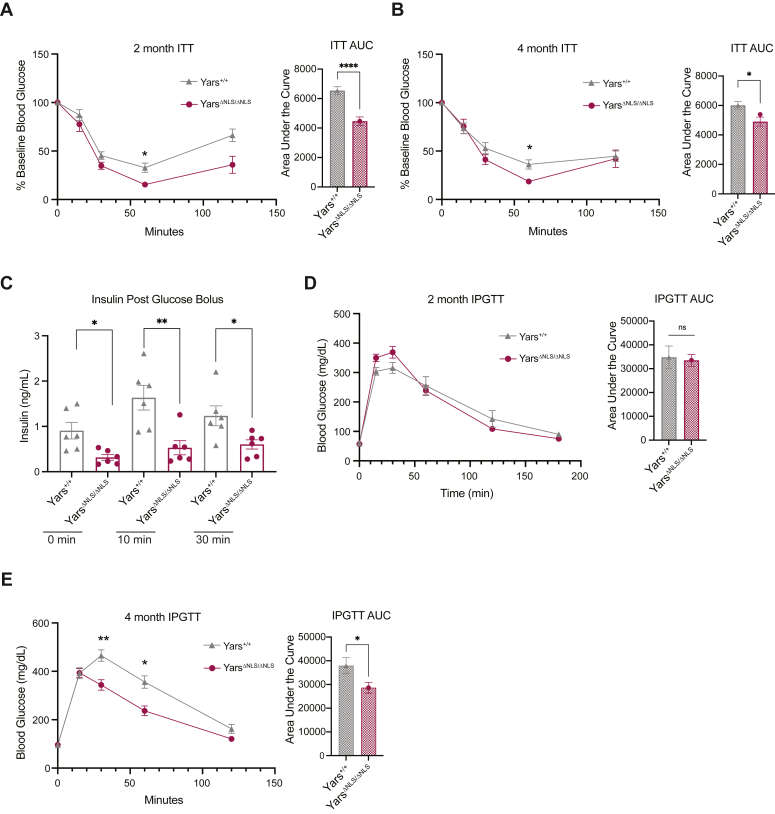
***Yars***^**ΔNLS**^**mice display an enhanced insulin sensitivity, decreased circulatory insulin, and improved glucose handling accompanying the smaller body weight.***A*, insulin tolerance test (ITT) of 2-month-old male mice after fasting for 6 h and injected intraperitoneally with insulin (1 U/kg). Mice below detection (<20 mg/dl) marked as 20 mg/dl. Analyzed by repeated measures two-way ANOVA with Sidak’s analysis. Area under the curve (AUC) calculated and analyzed by unpaired Welch’s *t* test. *Yars*^*+/+*^ N = 10, *Yars*^*ΔNLS/ΔNLS*^ N = 9. *B*, insulin tolerance test (ITT) of 4-month-old male mice after fasting for 6 h and injected intraperitoneally with insulin (1 U/kg). Mice below detection (<20 mg/dl) marked as 20 mg/dl. Analyzed by repeated measures two-way ANOVA with Sidak’s analysis. Area under the curve (AUC) calculated and analyzed by unpaired Welch’s *t* test. *Yars*^*+/+*^ N = 9, *Yars*^*ΔNLS/ΔNLS*^ N = 8. *C*, plasma insulin at 0-, 10-, and 30-min post glucose bolus (2 g/kg) in 3–4-month-old male mice. Mice fasted 6 h before injection. Repeated measures two-way ANOVA with Tukey’s comparison analysis. *Yars*^*+/+*^ N = 6, *Yars*^*ΔNLS/ΔNLS*^ N = 6. *D*, blood glucose levels during an intraperitoneal glucose tolerance test (IPGTT) of 2-month-old male mice after 16 h fast and injected with glucose (2 g/kg). Analyzed by repeated measures two-way ANOVA with Sidak’s analysis. Area under the curve (AUC) calculated and analyzed by unpaired Welch’s *t* test. *Yars*^*+/+*^ N = 8, *Yars*^*ΔNLS/ΔNLS*^ N = 9. *E*, blood glucose levels during an IPGTT of 4-month-old male mice after 16 h fast and injected with glucose (2 g/kg). Analyzed by repeated measures two-way ANOVA with Sidak’s analysis. Area under the curve (AUC) calculated and analyzed by unpaired Welch’s *t* test. *Yars*^*+/+*^ N = 9, *Yars*^*ΔNLS/ΔNLS*^ N = 8.

It is well established that chronic elevated ROS contributes to insulin resistance (40), whereas low levels or acute generation of ROS from physiological stimuli, such as hormones, enhances insulin sensitivity (41, 42, 43, 44). It is also evident that decreased body weight especially from fat mass leads to improved insulin sensitivity (45, 46). Therefore, we set out to investigate how the increased ROS and smaller body weight of *Yars*^ΔNLS^ mice affects insulin sensitivity. At 2 and 4 months, an insulin tolerance test (ITT) was performed, and the *Yars*^ΔNLS^ mice had an increased insulin sensitivity (Figs. 3, *A* and *B* and S3*A*). No substantial difference in plasma glucagon was found that would explain the insulin sensitivity (Fig. S3*B*). Considering the enhanced insulin sensitivity and reduced adiposity, insulin, another key metabolic hormone that promotes adipose tissue expansion through effects on lipogenesis and lipolysis was examined (47). Significant reductions in plasma insulin, with and without glucose stimulation, in both sexes were observed (Figs. 3*C* and S3*C*). Similarly to insulin sensitivity, glucose homeostasis typically improves as body weight and fat mass decreases (45, 46). Indeed, an intraperitoneal glucose tolerance test (GTT) performed at 2 months indicated that despite reduced insulin, the *Yars*^ΔNLS^ mice were able to maintain their glucose homeostasis (Figs. 3*D* and S3*D*). At 4 months in males, coinciding with significantly reduced adiposity, an improvement in glucose handling was observed (Fig. 3*E*). However, using an oral glucose bolus GTT, glucose tolerance in *Yars*^ΔNLS^ males seemed like the WT animals (Fig. S3*E*). Therefore, despite the decrease in circulatory insulin levels, glucose homeostasis was maintained and possibly improved in the *Yars*^ΔNLS^ mice suggesting an overall improvement in insulin action and glucose metabolism.

To exclude the possibility that the reduction in insulin was a pancreatic islet defect, we measured the total insulin content of the pancreas (Fig. S3*F*) and found no discernible difference. We also examined *ex vivo* the insulin secretion capacity of *Yars*^ΔNLS^ primary islets, and no defect in glucose stimulated insulin secretion was observed (Fig. S3*G*). Taken together, this implies the increased insulin sensitivity exhibited in the *Yars*^ΔNLS^ mice resulted in a reduced demand for circulating insulin.

### Yars^ΔNLS^ mice exhibit mild variations in insulin signaling of target tissues

Insulin signaling is mediated by the insulin receptor (IR) expressed in target tissues. The insulin binding to the α subunit of the receptor triggers a conformational change, leading to autophosphorylation of specific tyrosine residues on the β subunit of the receptor (48). Other signal protein partners, such as IRS-1, are recruited and then phosphorylated (48). Two major pathways are then activated that regulate metabolism and mitogenesis: PI3K/Akt and Ras/mitogen-activated protein kinase, respectively (48). To further understand the enhanced insulin sensitivity in *Yars*^ΔNLS^ mice, we assessed insulin signaling in target tissues, including skeletal muscle, epididymal white adipose (eWAT), and liver. Mice were fasted following administration of insulin or PBS, control, and tissues were dissected for Western blot analysis. The IR subunit β (IR-β) expression level appeared increased in *Yars*^ΔNLS^ eWAT but not in muscle or liver (Fig. S4, *A*–*C*). However, IR activation by insulin, as indicated by phosphorylation of IR-β, was significantly enhanced in *Yars*^ΔNLS^ muscle than WT, and liver followed a similar trend (Fig. S4, *B* and *C*). But for eWAT, IR activation was significantly increased for both genotypes (Fig. S4*A*). For all tissues, PI3K/Akt signaling as indicated by Akt^Ser473^ phosphorylation was significantly increased after insulin stimulation, but no significant differences were found in *Yars*^ΔNLS^ compared with the WT animals (Fig. S4, *A*–*C*).

As oxidative stress can influence insulin signaling, we analyzed lipid peroxidation as a marker of ROS (40, 41, 43). We observed an increased accumulation of 4-HNE protein adducts in the *Yars*^ΔNLS^ eWAT stimulated by insulin (Fig. S4*A*), but not in muscle and liver (Fig. S4, *B* and *C*). IGF-1, a hormone structurally similar to insulin, mediates growth and metabolism and can also activate the insulin receptor (49); therefore, we examined serum IGF-1 levels and saw no difference (Fig. S4*D*), suggesting IGF-1 is not playing a role in *Yars*^ΔNLS^ metabolism.

Overall, our observations point to mild changes at the expression or activation of IR, possibly with tissue-dependent effects. The increased oxidative stress upon insulin stimulation found in eWAT, but not in muscle and liver, may suggest a tissue specificity that is related to the increased fat oxidation and the lack of adipose storage. But the direct connections between them are unclear and require further investigation.

### Transcriptomic analysis reveals tissue-specific changes in the *Yars*^ΔNLS^ mice

To further understand tissue-specific impacts of nuclear TyrRS, we performed a bulk RNA-Seq experiment using three-month-old female mice. We compared eWAT and liver samples of *Yars*^ΔNLS^ *versus* the WT mice. The number of significantly dysregulated genes in the eWAT samples (112; 37 upregulated and 75 downregulated) was more than that in the liver tissues (41; 18 upregulated and 23 downregulated) (Fig. S5*A* and Table S1). Remarkably, about one third and one fourth of the dysregulated genes in eWAT and liver, respectively, were transcription factors (Fig. S5*B*), consistent with the established roles of nuclear TyrRS in transcriptional regulation (10, 11, 12). Only four genes were dysregulated in both tissues, including two transcription factors Tsc22d3 and Cebpd (Fig. S5*B*). No obvious correlation in overall gene fold change was observed between the two tissues (Fig. S5*C*). Based on gene ontology analysis, the dysregulated genes mostly impacted cell death, metabolic processes, and stress response in eWAT (Fig. S5*D*). In contrast, dysregulated genes in the liver were centered around biological processes related to cellular and organ system development, particularly toward blood cells (Fig. S5*D*), which may correlate with the smaller stature of *Yars*^ΔNLS^ mice. The analyses highlight the tissue-specific impact of nuclear TyrRS deficiency on transcription.

### Hearing loss observed in the *Yars*^ΔNLS^ mice

Lastly, we wanted to assess if other major body systems like the nervous system were affected in the *Yars*^ΔNLS^ mice. Nuclear TyrRS was shown to be involved in peripheral neuropathy, and hearing impairments were found in patients with biallelic mutations in *YARS1* (25, 27, 28, 34). The acoustic startle response test is commonly used to measure the intrinsic properties from the central nervous system, such as sensory gating, fear, and even anxiety-like behavior (50). The test can also probe the peripheral nervous system because its function is essential for signal transmission of the central nervous system to the muscle to elicit the startle. In both *Yars*^ΔNLS^ sexes, there was a significant reduction in startle response (Fig. S6*A*). We proposed there was a deficit in the neuromuscular reflex or upstream in the auditory system itself. To evaluate potential neuromuscular defects, the hindlimb extension was scored at three age points, and no gross differences were found (Fig. S6*B*). In addition, electrophysiology analysis revealed normal compound muscle action potential (CMAP) and repetitive nerve stimulation (RNS) of the sciatic nerve, suggesting intact neuromuscular reflex (Fig. S6, *C* and *D*).

**Figure 4:**
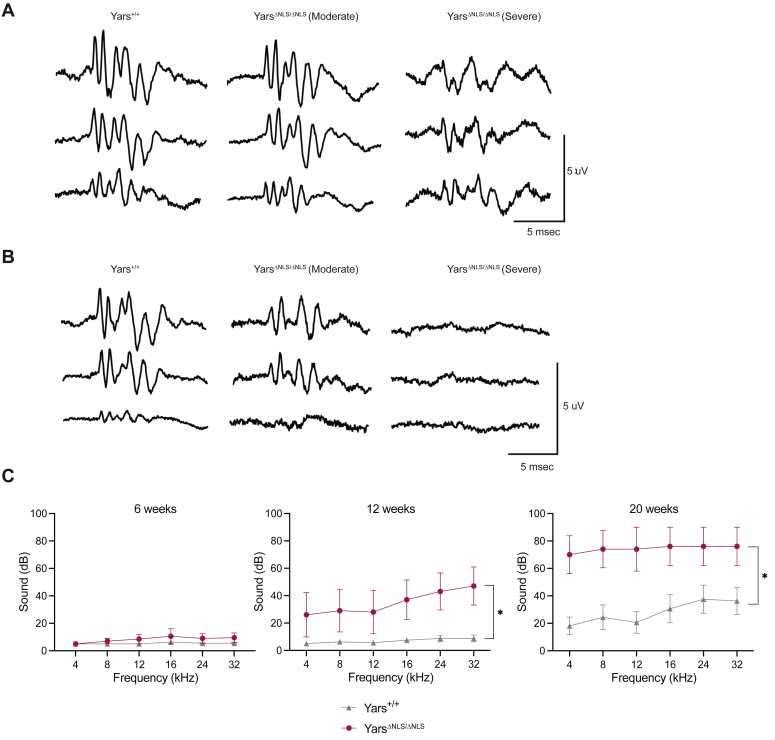
**Progressive hearing loss in the *Yars***^**ΔNLS**^**mice confirmed by auditory thresholds.***A*, representative auditory brainstem response (ABR) waveforms of male *Yars*^*+/+*^ and *Yars*^*ΔNLS/ΔNLS*^ mice at 6 months where mutants have variation between mice in severity. *B*, representative auditory brainstem response (ABR) waveforms of female WT and Yars^ΔNLS^ mice at 6 months where *Yars*^ΔNLS^ has variation between mice in severity. *C*, ABR thresholds from 4 to 32 kHz of both male and female mice at the indicated age point. (6, 12, and 20 weeks). Mice thresholds undetectable, marked as 90 dB. Repeated measures two-way ANOVA with Sidak’s analysis. 6 weeks: *Yars*^*+/+*^ N = 11, *Yars*^*ΔNLS/ΔNLS*^ N = 10; 12 and 20 weeks: *Yars*^*+/+*^ N = 8, *Yars*^*ΔNLS/ΔNLS*^ N = 5.

Therefore, we set out to investigate if *Yars*^ΔNLS^ mice had hearing abnormalities because the startle test relies on the mice responding to the auditory cue. We assessed their auditory activity by performing the brainstem auditory evoked potential (BAEP) response using tone pips of 1, 3, and 15 kHz at different sound pressure levels (SPLs) from 0 to 50 dB. In both *Yars*^ΔNLS^ mice sexes, the BAEP traces indicated a defect of variable penetrance in the auditory pathway (Fig. 4, *A* and *B*). *Yars*^ΔNLS^ mice exhibited moderate to severe decrease in amplitude in both sexes especially at peaks 1 to 3 with potential signal propagation and asynchronicity characteristics (Fig. 4, *A* and *B* and S7*A*). There was no peak or interpeak latency dysfunctions in either *Yars*^ΔNLS^ sex (Fig. 4, *A* and *B*, quantification not shown). After performing an auditory threshold, hearing loss was confirmed (Fig. S7*B*).

This study was conducted at 6 months, when age-related hearing loss occurs, especially in the C57BL6/J background (51, 52). To understand the onset of the hearing loss, auditory thresholds were conducted at 6, 12, and 20 weeks from 4 to 32 kHz. The *Yars*^ΔNLS^ mice exhibited no hearing loss at 6 weeks; however, substantial hearing loss was found at 12 weeks and progressively worsened at 20 weeks (Fig. 4*C*). In contrast, normal hearing was maintained in WT C57BL6/J animals at 12 weeks but started to deteriorate at 20 weeks (Fig. 4*C*). Therefore, the *Yars*^ΔNLS^ mutation triggers or accelerates a progressive form of hearing loss, which is not present from development.

## Discussion

The aaRS family are indispensable for protein synthesis across all domains of life for their canonical enzymatic function in charging tRNA. Yet, as organismal complexity increased the role of aaRS encompasses diverse regulatory functions, including some in the nucleus (4, 53, 54). However, due to the necessity of aaRS enzymes, it is challenging to dissect the impact of regulatory functions from the enzymatic. The challenge is especially true for TyrRS where the NLS is a dual-function motif also involved in tRNA binding (Fig. 1*A*), making it impossible to fully ablate the nuclear localization without substantially impacting tRNA aminoacylation. In an attempt to separate out the impact of nuclear TyrRS, we structurally and experimentally surveyed the NLS and selected a variant ^242-^NNKLNK^-247^ to maximally disrupt nuclear import, while minimally impacting tRNA charging (14). Indeed, while there is some residual nuclear TyrRS in the *Yars*^ΔNLS^ cells (Fig. 1*C*), a mild impact of the NLS mutant on cognate tRNA charging was also observed (Figs. 1*E* and S1*D*). However, the defect did not cause a reduction in global translation (Fig. S1*B*). Rather, new protein synthesis increased in *Yars*^ΔNLS^ MEFs, presumably due to general stress response pathways (55, 56) or the specific loss of nuclear TyrRS transcriptional repression on translation genes (12).

Previous studies have indicated nuclear translocation of TyrRS as a stress response (11, 12), and TyrRS nuclear deficiency exacerbates H_2_O_2_-induced cell death (12). Interestingly, the *Yars*^ΔNLS^ MEFs exhibit an enhanced mitochondrial respiration and increased ROS production even at normal cell culture conditions without the application of oxidative stress (Fig. S1, *B* and *C*), suggesting TyrRS nuclear deficiency not only makes cells more sensitive to stress conditions but also induces stress itself. An altered stress response can also lead to metabolic changes (20). Indeed, *Yars*^ΔNLS^ mice have a smaller stature but consume more food, are less active, and have enhanced metabolic rates (Fig. 2, *A*, *B*, and *D* and Table 1). This indicates inefficiencies in their energy consumption *versus* energy output. As a result, mutant mice, especially the males, are unable to accumulate fat (Fig. 2*C*). Interestingly, in female *Yars*^ΔNLS^ adipose tissue, “stress response” is a top impacted biological process and the dysregulated genes that contribute to this gene ontology term include an array of genes from adipokines (*e.g.*, Adipoq) to transcriptional regulators (*e.g.*, Atf3, Gadd45a, Myc) (Fig. S5*D*). This suggests not only are the *Yars*^ΔNLS^ MEF cells more prone to oxidative stress, but there are transcriptional changes at the tissue level that illustrates the role of nuclear TyrRS in stress response.

Fat storage is a vital physiological process, serving as an essential energy reserve, contributing to metabolic regulation, and a measure of overall health. In mammals like humans, white adipose tissue stores excess energy mainly in the form of triglycerides, providing a readily available source of fuel during times of scarcity. While excessive fat accumulation can lead to health complications such as obesity and metabolic disorders, maintaining a healthy level of fat storage is crucial for sustaining energy balance and supporting physiological processes like hormonal regulation because it is a major secretory organ (57, 58). Fat storage in vertebrates relies on white adipose tissue, whereas insects have fat bodies that resemble this adipose tissue (57). Considering the NLS of TyrRS is conserved from insects to humans, we speculate that the nuclear translocation of TyrRS was acquired during evolution to support, at least in part, a function that facilitates efficient energy storage in the form of white adipose tissue. Interestingly, “regulation of cell death” is the top biological process impacted in female *Yars*^ΔNLS^ adipose tissue (Fig. S5*D*), suggesting nuclear TyrRS plays a role in the maintenance of fat storage by inhibiting adipocyte apoptosis. Subsequent investigation is needed to understand how nuclear TyrRS protects adipocytes against apoptosis, in addition to a transcriptomic analysis with male animals that have a more dramatic fat difference.

The reduction in adiposity likely led to the enhanced insulin sensitivity that coincided with a reduced insulin demand and normal glycemic levels. This improved glucose handling, and insulin sensitivity has been associated with mouse models of increased exercise (59), calorie restriction diet (60), *etc.* (46) To explore the enhanced insulin sensitivity and its potential tissue specificity, we assessed the insulin signaling pathway in target tissues *ex vivo* but overall did not find a significant difference in *Yars*^ΔNLS^ mice (Fig. S4, *A*–*C*). Potentially we do not see more of an effect because these mice were treated with supraphysiological insulin levels, therefore possibly masking the nuances at physiological conditions. Therefore, an *in vivo* insulin action experiment using the hyperinsulinemic-euglycemic clamp technique should be considered in the future. The tissue-specific effects of insulin sensitivity are an important question still underway.

The increased fat oxidation of *Yars*^ΔNLS^ mice may lead to enhanced mitochondrial activity. We showed an enhanced mitochondrial respiration in *Yars*^ΔNLS^ MEFs (Fig. S1*C*), but future work should further evaluate mitochondrial activity in different tissues, especially adipose tissues. Increased fat oxidation typically results in lower circulating free fatty acids (FFAs) levels as they are utilized for energy in the mitochondria. However, we do not see reduced FFAs in *Yars*^ΔNLS^ mice (Fig. S2*D*), rather we see a reduction in insulin (Figs. 3*C* and S3*C*). Presumably, as insulin normally suppresses lipolysis (47), the insulin reduction would lead to an increase in lipolysis, balancing the effect from increased fat oxidation on circulating FFAs.

Sensorineural hearing loss is associated with metabolic disorders (61, 62) and is seen in at least half of *YARS*1 multisystem disorder patients (25, 26, 28, 29). However, little is understood regarding their correlation. In type 2 diabetes, there is some evidence of cochlear structure changes especially to the stria vascularis that is important for blood flow and ion composition, as well as damage to the auditory nerve (63, 64). Generally, there is a lack of understanding of the relevant genes for hearing maintenance under a dysregulated metabolic state. Our study suggests nuclear TyrRS as an important regulatory protein for hearing. The *Yars*^ΔNLS^ mice had progressive hearing loss starting at 3 months when they already exhibited a metabolic dysregulation (Fig. 4*C*). The BAEP waveform analysis indicated decreased peak amplitude (Fig. 4, *A* and *B*), which hinted at the cochlea as the likely site of lesion. Analysis of the cochlear morphology especially the stria vascularis, organ of Corti, and neuronal bundle would help to specifically pinpoint the likely cell type responsible for the hearing loss. It is also not clear if the hearing loss is through a cell autonomous or nonautonomous mechanism of nuclear TyrRS. To fully explore the hearing loss, a cochlear-specific *Yars*^ΔNLS^ mouse, ideally not in a C57BL6/J background, would help determine if the hearing loss is an independent phenomenon or secondary to metabolic dysregulation.

Nuclear TyrRS has been implicated in the peripheral neuropathy, CMT disease, where potentially gain of function mutations of TyrRS dysregulate transcription; however, if these mutants are excluded from the nucleus, the transcriptional dysregulation and neuromuscular phenotypes were restored in a *Drosophila* CMT model (34). We assessed motor function in the *Yars*^ΔNLS^ mice and observed no obvious neuromuscular problems (Fig. S6, *B*–*D*), highlighting that our nuclear deficiency is not like the toxic gain of function seen in CMT; rather, it is a loss of nuclear function, which does not obviously affect the peripheral nervous system.

The lack of gross neurological effects also distinguishes the *Yars*^ΔNLS^ mice from patients with *YARS1* biallelic mutations. This may reflect the differing severity of aminoacylation defects induced by the different mutations. Nonetheless, the fact that both the *Yars*^ΔNLS^ mouse model and the patients with *YARS1* biallelic mutations have common metabolic signatures, affecting insulin, lipids, and hearing, suggests that the noncanonical, regulatory functions of TyrRS could be an important factor contributing to the *YARS1* disease pathology.

In this study, we have successfully established the first murine model designed to elucidate the nuclear functions of an aaRS. The distinct metabolic phenotypes observed in the absence of nuclear TyrRS emphasize its pivotal role in the maintenance of metabolic homeostasis, ensuring effective energy storage in the form of adipose tissue. Considering the high energy demands of protein synthesis, which is among the most resource-intensive cellular processes (65), the precise coordination of protein production with energy management is essential for cellular efficiency. The adaptive movement of a key translation factor to the nucleus in response to environmental cues emerges as a crucial mechanism for regulating this equilibrium, ultimately conferring an evolutionary advantage to the organism.

## Experimental procedures

### Animal models

A targeting vector containing exon 7 of the *Yars1* gene (that contains the NLS sequence) mutated from ^242^KKKLKK^247^ to ^242^NNKLNK^247^, with 3′ and 5′ homology arms, was electroporated into mouse ES cells. Correctly targeted clones were screened by PCR and used for blastocyst injection. After screening and confirmation of germline transmission, knock-in mice were bred with FLP transgenic mice to delete FRT-flanked Neomycin, thereby generating ΔNLS-Neo-Out strain. mRNA expression of the mutant allele was confirmed through RT-PCR, and functional disruption of nuclear import was confirmed by Western blot. These F0 mice were crossed with C57BL/6J mice to obtain *Yars*^+/ΔNLS^ mice that were used for breeding thereafter. Female and male mice were used for breeding from 3 to 8 months of age. Tissue from animals was saved for genotyping. DNA was extracted using Lucigen QuickExtract (VWR, 76081-766), and the knock-in allele were detected by PCR amplification with the primers below and visualized by a 2% agarose gel:

Forward: ATAGGTTCTTCCTGCAGGGAC

Reverse: TCTGCCTCTAGTCTCATTTTGGATTC

Mouse experiments were approved by and conducted in accordance with the guidelines of The Scripps Research Institute IACUC.

### Primary cell culture

Yars^ΔNLS/ΔNLS^ primary MEFs were isolated from heterozygous, *Yars*^*+/ΔNLS*^, mice as previously described (66). Briefly, embryos were collected between E12.5 – E14.5 from pregnant (*Yars*^*+/ΔNLS*^ *x Yars*^*+/ΔNLS*^) females that were dissected out of uterine horns into PBS with 1% penicillin/streptomycin. The embryos were separated from the placenta, and the head and organs were removed, then remaining blood rinsed off. The cells were isolated from trypsinized embryonic tissue. Cells from each embryo were plated in a 10 cm dish with media containing Dulbecco’s modified Eagle’s medium (DMEM) with Glutamax (Thermo Fisher Scientific), 10% heat-inactivated embryonic stem cell fetal bovine serum (Thermo Fisher Scientific, 16141079), and 1% penicillin/streptomycin (Thermo Fisher Scientific). Primary MEFs were maintained in a 37 °C incubator at 5% CO_2_, and all experiments were conducted at passages 1 to 3.

### Nuclear-cytoplasmic fractionation

8 × 10^6^ MEF cells seeded on 10 cm dishes were washed with Dulbecco's Phosphate-Buffered Saline (DPBS), scraped in 1 ml DPBS, and 200 μl were taken out for whole cell lysate (lysed in 2X RIPA). The remaining cells were pelleted, placed in cell fractionation buffer (20 mM Hepes, pH 7.4, 10 mM KCl, 2 mM MgCl_2_, 2 mM EDTA, 0.7% NP-40, protease inhibitor) and incubated on ice for 20 min. Cells were passed five times through a 27 g needle and centrifuged for 5 min at 5000*g* to separate nucleus and cytosol. The nuclear pellet was washed two times with wash buffer containing 20 mM Hepes pH 7.4, 10 mM KCl, 2 mM MgCl_2_, 2 mM EDTA, and 0.5% NP-40 and finally solubilized in 100 μl 2X RIPA. Whole cell lysate, cytosolic, and nuclear fractions were centrifuged at 16,000*g* for 15 min, the supernatant was mixed with 4X LDS loading buffer, and boiled for 5 min. Samples were loaded on an SDS gel and analyzed by Western blot analysis.

### Cellular ROS assay

Cells were plated in 24-well tissue culture–treated plates the day before. CM-H2DCFDA (Invitrogen, C6827) was freshly dissolved in DMSO. Cells were incubated in 5 μM CM-H2DCFDA for 30 min with or without 10 μM H_2_O_2_ and then immediately run on a NovoCyte 3000 (Acea) using excitation 488 nm and emission 530 nm channel (FITC channel). Cytometric analysis was performed using FlowJo software (BD Biosciences). Unstained control and positive control: cells treated with 100 μM H_2_O_2_.

### Puromycin incorporation assay

MEFs were seeded in a 6-well plate at 200,000 cells per well. The next day, cells were treated with 10 μg/ml of puromycin for 30 min, washed with DPBS, and chased with regular growth medium for 1 h. Cells were washed with cold DPBS, suspended in 1X RIPA buffer with protease inhibitors, and analyzed by Western blot using a puromycin antibody (1:3000, Millipore, MABE343) with *α*-Tubulin (1:3000, CST) used as a loading control.

### Mitochondrial respiration

Respiration analysis using a Seahorse XFe96 analyzer. MEFs were seeded 1 day prior on 96-well Seahorse plates (Agilent) coated with CellTak (Corning). The next day, cells were equilibrated in Seahorse media containing DMEM with no phenol red nor bicarbonate, glucose, glutamate, sodium pyruvate, and 1% embryonic stem cell fetal bovine serum (FBS) (Invitrogen). The following were loaded sequentially: 2 μM oligomycin, 0.5 μM FCCP, and 1 μM Rotenone/Antimycin. For normalizations, equal numbers of cells per condition were seeded followed by a BCA analysis after the stress test.

### Acidic gel northern blot

Determination of the levels of acylated tRNA *in vivo* was performed as previously described (67). Briefly, total RNA was extracted from cells using Trizol (following manufacturer guidelines), and the RNA pellet was dissolved in 50 mM sodium acetate pH 5.0. To deacylate the tRNA, the pellet was dissolved in 0.2 M Tris–HCl, pH 9.5 and incubated for 1 h at 37 °C, then precipitated again using isopropanol, and resuspended in 50 mM sodium acetate pH 5.0 The RNA was mixed with equal volumes of sample buffer (0.1 M sodium acetate pH 5.0, 8 M urea, 0.05% bromophenol blue, 0.05% xylene cyanol). RNA (3 μg) was separated on a 0.4 mm thick 6.5% polyacrylamide gel containing 8 M urea in 0.1 M sodium acetate buffer pH 5.0 using 42 × 20 cm glass. Electrophoresis was performed in the cold room at 500 V until the bromophenol blue dye reached 27 cm (∼18 h). The portion of the gel between the xylene cyanol and bromophenol blue bands was excised and transferred onto a Hybond N+ membrane using a Semi-dry Transfer Cell (Biorad) at 5 V/150 mA for 60 min soaked in 0.5X TBE as transfer buffer. The membrane was UV crosslinked (2 × 0.12 J) and then pre-hybridized with hybridization buffer containing 20 mM sodium phosphate pH 7.2, 300 mM NaCl, 1% SDS. The blot was then hybridized with 5′ end biotinylated labeled DNA oligonucleotide probes (IDT) at 60 °C in hybridization buffer overnight. Blots were washed twice for 15 min with 20 mM sodium phosphate pH 7.2, 300 mM NaCl, 0.1% SDS, and 2 mM EDTA at room temperature followed by an incubation with streptavidin-HRP antibody (BioLegend, 1:5000) for 30 min at 37 °C. The blots were then washed three times for 5 min at room temperature, dried with filter paper, and incubated with chemiluminescent substrate (Prometheus ProSignal Dura, Genesee Scientific) for 3 min and imaged (Biorad, ChemiDoc). Quantification was performed on Biorad’s Image Lab v6 software. To strip the blot, blots were incubated at 80 °C for 1 h in hybridization buffer.

Probes:

tRNA^Tyr(GUA)^:TCCTTCGAGCCGGANTCGAACCAGCGACCTAAGGATCTACAGTCCTCCGCTCTACCANCTGAGCTATCGAAGG

tRNA^Ser(CGA)^:GCTGTGAGCAGGATTTGAACCTGCGCGGGGARACCCCATTGGATTTCGAGTCCAACGCCTTAACCACTCGGCCATCACAG.

### EchoMRI

The EchoMRI 3-in-1 instrument (EchoMRI LLC) is a quantitative nuclear magnetic resonance imaging system for whole body composition analysis of unanesthetized small animals, and quantitative nuclear magnetic resonance body composition analysis with EchoMRI instrumentation has been proposed to be “gold standard” methodology for metabolic studies in the mouse (68, 69). Following calibration, each mouse was put in a holder and placed into the EchoMRI chamber and lean mass, fat mass, and water mass were calculated.

### Comprehensive laboratory animal monitoring system

Indirect calorimetry was performed in acclimated, single-housed mice using a computer-controlled, open-circuit system (Oxymax System) that is part of an integrated CLAMS (Columbus Instruments). Testing occurred in clear respiratory chambers (20 × 10 × 12.5 cm) equipped with a sipper tube delivering water, food tray connected to a balance, and 16 photobeams situated in rows at 0.5 in intervals to detect motor activity along the x- and z-axes. Room air was passed through chambers at a flow rate of 0.5 L/min. Exhaust air from each chamber was sampled at 15-min intervals for 1 min. Sample air was sequentially passed through O_2_ and CO_2_ sensors (Columbus Instruments) for determination of O_2_ and CO_2_ content, from which measures of oxygen consumption (VO_2_) and carbon dioxide production (VCO_2_) were estimated. RERs were calculated as the ratio of carbon dioxide production (VCO_2_) to oxygen consumption (VO_2_) and all measures were corrected for effective metabolic mass by using each mouse’s lean mass obtained from the EchoMRI test. Energy expenditure and fat oxidation was calculated with the following equation from Bruss *et. al.* EE (kcal/h/kg) = [(3.815 + 1.232 × RER) x VO_2_] x 1000; FA oxidation = EE x (1 – RER/0.3) (70). Carbohydrate oxidation was calculated with the following equation from Loh *et. al.* Carb oxidation = 4.585 VCO_2_ - 3.226 VO_2_ (41).

### Plasma and serum measurements

Blood was obtained by tail vein collection. Plasma insulin was quantified using the Ultra Sensitive Mouse Insulin ELISA (Crystal Chem, 90080). Plasma glucagon was quantified using the Glucagon 10 μl ELISA (Mercodia, 10-1281-01). Plasma IGF-1 was quantified using the Mouse IGF-1 ELISA (Crystal Chem, 80574). Serum triglycerides were quantified using the LabAssay Triglycerides kit (Fujifilm Wako Chemicals, 632-50991). Serum-free fatty acids were measured using the Free Fatty Acid Quantitation Kit (Sigma Aldrich, MAK044-1). Serum cholesterol was quantified using the Cholesterol E kit (Wako Diagnostics, 999-02601). All kits followed the manufacturer’s guidelines.

### Histological analyses

Epididymal adipose and liver tissues were fixed in 4% paraformaldehyde, embedded in paraffin, sectioned at 10 μm, and stained with hematoxylin and eosin.

### Oil red O

For MEFs differentiated into adipocytes, MEFs at passage 1 were grown to confluency in regular growth medium and then switched to an adipocyte differentiation medium containing advanced DMEM-F12 (Gibco), 10% ES-FBS (Gibco), GlutaMax, Penicillin/streptomycin, 0.2 mM L-ascorbic acid, 1 μM dexamethasone that was changed every 3 days for 14 days. Negative differentiation control is the same MEFs in regular growth media. Cells were imaged on a light microscope and at day 14, cells were fixed in 4% paraformaldehyde, washed with 60% isopropanol, stained with oil red O for 1 h, washed three times with H_2_O, and 1 ml 100% isopropanol was added while shaking. Then 200 μl was transferred to a 96 well and absorbance read at 505 nm.

### GTTs and ITTs

For GTTs, mice were fasted overnight for 16 h prior to administration of a 2 g/kg glucose bolus *via* oral gavage or intraperitoneal injection, as noted in the text. For ITTs, mice were fasted for 6 h prior to intraperitoneal injection of insulin (1 U/kg, Humulin R). Tail blood glucose levels were determined at the indicated time points using the Contour Next Blood Glucose Meter and Contour Next Test Strips.

### Insulin signaling

Male mice were age matched and fasted for 4 h prior to injections. Mice were randomly assigned PBS or insulin (1 U/kg, Humulin R) groups and then injected and sacrificed 15 min post-injection. Blood was washed away by a transcardiac perfusion with PBS: By fully exposing the heart and lung of the mouse from reflecting the sternum up with hemostat, the heart was secured at a steady position and a needle (27G) was inserted from the tip of the left ventricle at an angle approximately parallel to the midline of the heart and steadily advanced the needle until it entered the ascending aorta. Followed by a small incision on the right atrium, the PBS perfusion started at a constant speed of ∼1 ml/5 s by pushing the syringe. Perfusion stopped when the fluid flowing out was clear of blood. For adult mice, it took ∼10 ml DPBS. Then tissues were extracted and snap frozen in liquid N_2_ until further processing. Tissues were homogenized in 1%NP40/DPBS containing protease and phosphatase inhibitors using a mechanical homogenizer. Protein lysates were measured with a BCA kit (Thermo Fisher Scientific).

### Western blot assay

Lysates collected were fractionated by SDS-PAGE and transferred to nitrocellulose membranes using the iBlot 2 Dry Blotting System (Thermo Fisher Scientific). Membranes were blocked for 1 h with Tris Buffered Saline with Tween 20 (TBST) containing 5% nonfat dry milk or 5% BSA TBST. The following primary antibodies were diluted in 1% milk in TBST or 5% BSA in TBST prior to usage at the indicated concentration overnight at 4 °C: Lamin A/C (1:3000, CST), *α*-Tubulin (1:3000, CST), β-Actin (1:3000, CST), Akt (1:2000, CST), phosphorylated Akt (1:1000, CST), IR-β (1:1000, CST), IR-β Y1146 (1:1000, CST), Puromycin (1:3000, Millipore MABE343), 4-HNE (1:1000, Alpha Diagnostics), and TyrRS (1:3000, homemade). After incubation with primary antibodies, the membranes were washed three times and incubated with HRP-conjugated anti-mouse or anti-rabbit secondary antibodies (1:8,000, Invitrogen) for 1 h, washed three times, and followed by detection with ECL substrate (Prometheus ProSignal Dura, Genesee Scientific). Western blots were visualized on a Bio-Rad ChemiDoc and analyzed with their Image Lab v6 software.

### Glucose-stimulated insulin secretion

Primary mouse islets were isolated, and 4 ml of collagenase P (Roche) was perfused into the pancreas at a concentration of 0.8 mg/ml through the common bile duct. The pancreas was then removed and dissociated at 37 °C for 13 min. Islets were separated using a gradient composed of HBSS and Histopaque (Sigma) layers. Purified islets were hand-picked using a dissection microscope. Islets were incubated overnight in RPMI 1640 supplemented with 8 mM glucose, 10% FBS, 2 mM L-glutamine, 100 U/ml Pen/Strep, 1 mM sodium pyruvate, 10 mM Hepes (Invitrogen). The following day, islets were washed and pre-incubated for 1 h in Krebs-Ringers-Bicarbonate-Hepes (KRBH) buffer (130 mM NaCl, 5 mM KCl, 1.2 mM CaCl_2_, 1.2 mM MgCl_2_, 1.2 mM KH_2_PO_4_, 20 mM HEPES pH 7.4, 25 mM NaHCO_3_, and 0.1% bovine serum albumin) supplemented with 2.8 mM glucose solution at 37 °C in 5% CO_2_. Afterward, groups of five islets that were size-matched between groups were transferred to a 96-well plate with KRBH solution containing low glucose (2.8 mM), high glucose (16.8 mM), or KCl (5 mM). After incubation for 1 h, the supernatant was collected, and islets were lysed overnight in a 20% hydrochloric acid: 80% ethanol solution. Insulin was then measured in supernatants and lysates using the Ultra Sensitive Mouse Insulin ELISA (Crystal Chem, 90080).

### Bulk RNA-Seq

Approximately, 12-week-old female mice were used for tissue isolation. Total RNA was isolated using an RNeasy Plus Mini Kit (Qiagen) and at the Genomic Core of Scripps Research, they were prepared into RNAseq libraries using the NEBNext Ultra II Directional RNA Library Prep Kit for Illumina following manufacturer’s recommended protocol. Briefly, for each sample, 100 to 500 ng total RNA was polyA selected, converted to double stranded cDNA followed by fragmentation, and ligation of sequencing adapters. The libraries were then PCR amplified 12 cycles using barcoded PCR primers, purified, and size selected using AMPure XP Beads before loading onto an Element Aviti for 2 × 75 base paired-end sequencing. The kallisto tool (71) was used to summarize the expression level of the individual genes from the RNA-seq fastq files, based on the Ensembl mouse cDNA annotation (GRCm39). The edgeR tool (72) was applied to identify the differentially expressed genes. Briefly, the TMM method was applied for read count normalization, while the likelihood ratio test was employed for differentially expressed gene identification. The genes with false discovery rate < 0.1 and fold change > 1.5 were considered differentially expressed. Functional annotation terms were determined using ShinyGO Version 0.80.0 (73).

### Brainstem auditory evoked potentials

Mice were maintained under controlled conditions (21 °C ± 2 °C; relative humidity 50%-60%; 12 h light-dark cycle, lights on 6:00 AM), throughout the study with free access to food and water. For the implantation of recording electrodes, mice were anesthetized using isoflurane (1–1.5%) and their heads were placed in a stereotaxic apparatus. Head hair was shaved off and the incision site was prepared with ethanol and betadine. An incision was made, and the skull was exposed and cleaned. Three stainless steel screw electrodes were inserted into the skull: one over the frontal bone (+2.0 mm anterior and +2.0 mm lateral to bregma). One more over the hippocampus (−2.0 mm posterior and +2.0 mm lateral to bregma) and a third over the cerebellum as a control for signal artifacts. Insulated leads from these electrodes were then soldered to a mini-connector that was cemented to the skull with dental acrylic. Wounds were sutured closed, topical antibiotic ointment was applied, mice were injected subcutaneously with flunixin, and the mice were recovered in a clean warm cage. Mice were monitored continuously during surgery and until fully recovered and then at least once per day until the experiments end. After surgery, mice were housed in individual cages and allowed a 1 week recovery before recordings.

For BAEPs, mice were anesthetized using isoflurane (1–1.5%) and then fitted with bilateral polyethylene ear tubes placed into the external auditory canal. A tube was attached to the central sound source (Biopac Systems, Inc. STM 100c), and a ‘Y’-connector was used to deliver the auditory stimulation in both ears. A computer program (Biopac Systems, Inc. MP 160) was used to generate the sound stimulus and to amplify and collect the data (Biopac Systems, Icn. ERS 1000). BAEPs were recorded to clicks of 10 μs duration, positive transient, from 0 to 85 dB SPL and presented 1024 times at 42.6/sec. BAEPs threshold was determined by an audiometry tested at 6, 12, and 20 weeks of age, using different frequencies (4, 8, 12, 16, 24, and 32 kHz) and different decibel levels (starting with 0 db and no more than 80 db SPL), for each frequency (74, 75). BAEP peak latencies and peak amplitudes were calculated using the Biopac Systems MP 160. Deafness/hearing impairments were determined by the absence of the BAEPs peaks.

### Acoustic startle reflex test

The acoustic startle response is a short-latency reflex that is mediated by a simple neural circuit and has been used to assess hearing (76). Startle is elicited by brief noise bursts between 90 and 120 dB. This test was performed using San Diego Instruments startle chambers (SR-Lab) which consist of nonrestrictive Plexiglas cylinders 5 cm in diameter resting on a Plexiglas platform in a ventilated chamber. High frequency speakers mounted 33 cm above the cylinders produce all acoustic stimuli, which are controlled by SR-LAB software. Piezoelectric accelerometers mounted under the cylinders transduce movements of the animal, which are digitized and stored by an interface and computer assembly. A 60 min test session was used, in which 40 ms pulses of 90, 95, 100, 105, 110, 115, and 120 dB white noise were presented multiple several times in a random order. Beginning at startling stimulus onset, 65 consecutive 1 ms readings were recorded to obtain the peak amplitude of the animal's startle response. The shapes of the startle response curves were used to make predictions about hearing in the mice.

### Hindlimb extension test

This quick assessment provides an index of neuromuscular deficit, with lower scores being associated with greater deficit (77). The mouse is held in the air by its tail for 10s and the position of the hindlimbs is scored as follows: 0 = hindlimbs tucked in, 1 = one leg tucked in and the other extended, and 2 = both legs extended.

### Muscle electromyography

For CMAP, mice were anesthetized at 2 L/min at 2% isoflurane in the anesthesia chamber and maintained anesthesia at 0.4 L/min at 2% using a nose cone. A rectal temperature probe was inserted to monitor temperature as mice were placed prone on a regulated heating plate (ML295/M Homeothermic Controller and Plate) set to an internal temperature of 37 °C, and rear limbs facing outwards and tail facing down were taped. Using a PowerLab 8/35 with FE231 Bio Amp, a ground electrode was placed just under the skin of right paw, a recording electrode was placed in the right rectus femoris leg muscle, and a reference electrode was placed just under the skin of left paw. To determine the optimal position of stimulator electrodes (TECAelite Disposable Monopolar Needles, Ref 902-DMF25-S) for sciatic nerve stimulation, the stimulator anode was inserted by the hip, beneath the skin, at a depth of approximately 18 mm and the stimulator cathode was inserted parallel to the hip approximately 5 mm from stimulator anode. Using LabChart Pro as the recording and controlling software, the best position for cathode lead was found by starting stimulation and pulling out stimulator cathode lead slowly. Once a signal appeared, the cathode lead position was adjusted for maximal amplitude. Finally, the current was adjusted to determine maximum amplitude at lowest current. This amplitude was reported as the CMAP in mV. To calculate RNS, a stimulation train of 10 impulses were carried out at 1, 4, 10, and 20 Hz at 125% of previously determined CMAP current. RNS was reported as the 10th impulse amplitude in percent, using the first impulse as reference for each frequency.

### Statistical analysis

GraphPad Prism 10 was used for data analysis and statistical significance was calculated using unpaired Student’s *t* test with Welch’s correction, one-way ANOVA or two-way ANOVA with specific tests indicated in figure legends. Statistically significant differences are indicated in figures legends. Error bars in figures indicate the SEM for the number of replicates (N), as indicated in the figure. *p* < 0.05(∗), *p* < 0.01(∗∗), *p* < 0.001(∗∗∗), and *p* < 0.0001(∗∗∗∗) were considered significant.

## Data availability

RNA-Seq data have been deposited and are publicly available at SRA (PRJNA1148973).

## Supporting information

This article contains supporting information.

## Conflict of interest

The authors declare that they have no conflicts of interest with the contents of this article.

## References

[1] Schimmel P.R., Söll D. (1979). Aminoacyl-tRNA synthetases: general features and recognition of transfer RNAs. Annu. Rev. Biochem..

[2] Han J.M., Jeong S.J., Park M.C., Kim G., Kwon N.H., Kim H.K. (2012). Leucyl-tRNA synthetase is an intracellular leucine sensor for the mTORC1-signaling pathway. Cell.

[3] Wakasugi K., Slike B.M., Hood J., Otani A., Ewalt K.L., Friedlander M. (2002). A human aminoacyl-tRNA synthetase as a regulator of angiogenesis. Proc. Natl. Acad. Sci. U. S. A..

[4] Shi Y., Xu X., Zhang Q., Fu G., Mo Z., Wang G.S. (2014). tRNA synthetase counteracts c-Myc to develop functional vasculature. Elife.

[5] Arif A., Jia J., Moodt R.A., DiCorleto P.E., Fox P.L. (2011). Phosphorylation of glutamyl-prolyl tRNA synthetase by cyclin-dependent kinase 5 dictates transcript-selective translational control. Proc. Natl. Acad. Sci. U. S. A..

[6] Shi Y., Liu Z., Zhang Q., Vallee I., Mo Z., Kishi S. (2020). Phosphorylation of seryl-tRNA synthetase by ATM/ATR is essential for hypoxia-induced angiogenesis. PLoS Biol..

[7] Wakasugi K., Nakano T., Morishima I. (2005). Oxidative stress-responsive intracellular regulation specific for the angiostatic form of human tryptophanyl-tRNA synthetase. Biochemistry.

[8] Wakasugi K., Schimmel P. (1999). Two distinct cytokines released from a human aminoacyl-tRNA synthetase. Science.

[9] Kanaji T., Vo M.N., Kanaji S., Zarpellon A., Shapiro R., Morodomi Y. (2018). Tyrosyl-tRNA synthetase stimulates thrombopoietin-independent hematopoiesis accelerating recovery from thrombocytopenia. Proc. Natl. Acad. Sci. U. S. A..

[10] Wei N., Shi Y., Truong L.N., Fisch K.M., Xu T., Gardiner E. (2014). Oxidative stress diverts tRNA synthetase to nucleus for protection against DNA damage. Mol. Cell.

[11] Cao X., Li C., Xiao S., Tang Y., Huang J., Zhao S. (2017). Acetylation promotes TyrRS nuclear translocation to prevent oxidative damage. Proc. Natl. Acad. Sci. U. S. A..

[12] Jones J.A., Wei N., Cui H., Shi Y., Fu G., Rauniyar N. (2023). Nuclear translocation of an aminoacyl-tRNA synthetase may mediate a chronic "integrated stress response". Cell Rep..

[13] Zhao R., Cai K., Yang J.J., Zhou Q., Cao W., Xiang J. (2023). Nuclear ATR lysine-tyrosylation protects against heart failure by activating DNA damage response. Cell Rep..

[14] Fu G., Xu T., Shi Y., Wei N., Yang X.L. (2012). tRNA-controlled nuclear import of a human tRNA synthetase. J. Biol. Chem..

[15] Thompson D.M., Parker R. (2009). Stressing out over tRNA cleavage. Cell.

[16] Huh D., Passarelli M.C., Gao J., Dusmatova S.N., Goin C., Fish L. (2021). A stress-induced tyrosine-tRNA depletion response mediates codon-based translational repression and growth suppression. EMBO J..

[17] Schwenzer H., Juhling F., Chu A., Pallett L.J., Baumert T.F., Maini M. (2019). Oxidative stress triggers selective tRNA retrograde transport in human cells during the integrated stress response. Cell Rep..

[18] Roberts C.K., Sindhu K.K. (2009). Oxidative stress and metabolic syndrome. Life Sci..

[19] Monserrat-Mesquida M., Quetglas-Llabrés M., Capó X., Bouzas C., Mateos D., Pons A. (2020). Metabolic syndrome is associated with oxidative stress and proinflammatory state. Antioxidants (Basel).

[20] Rani V., Deep G., Singh R.K., Palle K., Yadav U.C. (2016). Oxidative stress and metabolic disorders: pathogenesis and therapeutic strategies. Life Sci..

[21] Kishimoto-Urata M., Urata S., Fujimoto C., Yamasoba T. (2022). Role of oxidative stress and antioxidants in acquired inner ear disorders. Antioxidants (Basel).

[22] Sajish M., Schimmel P. (2015). A human tRNA synthetase is a potent PARP1-activating effector target for resveratrol. Nature.

[23] Sissler M., González-Serrano L.E., Westhof E. (2017). Recent advances in mitochondrial aminoacyl-tRNA synthetases and disease. Trends Mol. Med..

[24] Jiang L., Jones J., Yang X.L. (2020). Human diseases linked to cytoplasmic aminoacyl-tRNA synthetases. Enzymes.

[25] Tracewska-Siemiatkowska A., Haer-Wigman L., Bosch D.G.M., Nickerson D., Bamshad M.J., van de Vorst M. (2017). An expanded multi-organ disease phenotype associated with mutations in YARS. Genes (Basel).

[26] Nowaczyk M.J., Huang L., Tarnopolsky M., Schwartzentruber J., Majewski J., Bulman D.E. (2017). A novel multisystem disease associated with recessive mutations in the tyrosyl-tRNA synthetase (YARS) gene. Am. J. Med. Genet. A..

[27] Williams K.B., Brigatti K.W., Puffenberger E.G., Gonzaga-Jauregui C., Griffin L.B., Martinez E.D. (2019). Homozygosity for a mutation affecting the catalytic domain of tyrosyl-tRNA synthetase (YARS) causes multisystem disease. Hum. Mol. Genet..

[28] Averdunk L., Sticht H., Surowy H., Lüdecke H.J., Koch-Hogrebe M., Alsaif H.S. (2021). The recurrent missense mutation p.(Arg367Trp) in YARS1 causes a distinct neurodevelopmental phenotype. J. Mol. Med. (Berl).

[29] Zeiad R., Ferren E.C., Young D.D., De Lancy S.J., Dedousis D., Schillaci L.A. (2021). A novel homozygous missense mutation in the YARS gene: expanding the phenotype of YARS multisystem disease. J. Endocr. Soc..

[30] Mendes M.I., Green L.M.C., Bertini E., Tonduti D., Aiello C., Smith D. (2020). RARS1-related hypomyelinating leukodystrophy: expanding the spectrum. Ann. Clin. Transl. Neurol..

[31] Abbott J.A., Guth E., Kim C., Regan C., Siu V.M., Rupar C.A. (2017). The usher syndrome type IIIB Histidyl-tRNA synthetase mutation confers temperature sensitivity. Biochemistry.

[32] Xu Z., Lo W.S., Beck D.B., Schuch L.A., Olahova M., Kopajtich R. (2018). Bi-Allelic mutations in phe-tRNA synthetase associated with a multi-system pulmonary disease support non-translational function. Am. J. Hum. Genet..

[33] Jordanova A., Irobi J., Thomas F.P., Van Dijck P., Meerschaert K., Dewil M. (2006). Disrupted function and axonal distribution of mutant tyrosyl-tRNA synthetase in dominant intermediate Charcot-Marie-Tooth neuropathy. Nat. Genet..

[34] Bervoets S., Wei N., Erfurth M.L., Yusein-Myashkova S., Ermanoska B., Mateiu L. (2019). Transcriptional dysregulation by a nucleus-localized aminoacyl-tRNA synthetase associated with Charcot-Marie-Tooth neuropathy. Nat. Commun..

[35] Pan T. (2018). Modifications and functional genomics of human transfer RNA. Cell Res..

[36] Waldron A., Wilcox C., Francklyn C., Ebert A. (2019). Knock-down of histidyl-tRNA synthetase causes cell cycle arrest and apoptosis of neuronal progenitor cells *in vivo*. Front. Cell Dev. Biol..

[37] Zeng Q.Y., Zhang F., Zhang J.H., Hei Z., Li Z.H., Huang M.H. (2023). Loss of threonyl-tRNA synthetase-like protein Tarsl2 has little impact on protein synthesis but affects mouse development. J. Biol. Chem..

[38] Siekierska A., Stamberger H., Deconinck T., Oprescu S.N., Partoens M., Zhang Y. (2019). Biallelic VARS variants cause developmental encephalopathy with microcephaly that is recapitulated in vars knockout zebrafish. Nat. Commun..

[39] Xu P., Wang L., Peng H., Liu H., Liu H., Yuan Q. (2021). Disruption of Hars2 in cochlear hair cells causes progressive mitochondrial dysfunction and hearing loss in mice. Front. Cell Neurosci..

[40] Newsholme P., Cruzat V.F., Keane K.N., Carlessi R., de Bittencourt P.I. (2016). Molecular mechanisms of ROS production and oxidative stress in diabetes. Biochem. J..

[41] Loh K., Deng H., Fukushima A., Cai X., Boivin B., Galic S. (2009). Reactive oxygen species enhance insulin sensitivity. Cell Metab..

[42] Woo H.A., Yim S.H., Shin D.H., Kang D., Yu D.Y., Rhee S.G. (2010). Inactivation of peroxiredoxin I by phosphorylation allows localized H(2)O(2) accumulation for cell signaling. Cell.

[43] Iwakami S., Misu H., Takeda T., Sugimori M., Matsugo S., Kaneko S. (2011). Concentration-dependent dual effects of hydrogen peroxide on insulin signal transduction in H4IIEC hepatocytes. PLoS One.

[44] Goldstein B.J., Mahadev K., Wu X., Zhu L., Motoshima H. (2005). Role of insulin-induced reactive oxygen species in the insulin signaling pathway. Antioxid. Redox Signal..

[45] Krüger J., Brachs S., Trappiel M., Kintscher U., Meyborg H., Wellnhofer E. (2015). Enhanced insulin signaling in density-enhanced phosphatase-1 (DEP-1) knockout mice. Mol. Metab..

[46] Lin Z., Tian H., Lam K.S., Lin S., Hoo R.C., Konishi M. (2013). Adiponectin mediates the metabolic effects of FGF21 on glucose homeostasis and insulin sensitivity in mice. Cell Metab..

[47] Norton L., Shannon C., Gastaldelli A., DeFronzo R.A. (2022). Insulin: the master regulator of glucose metabolism. Metabolism.

[48] Boura-Halfon S., Zick Y. (2009). Phosphorylation of IRS proteins, insulin action, and insulin resistance. Am. J. Physiol. Endocrinol. Metab..

[49] Werner H. (2023). The IGF1 signaling pathway: from basic concepts to therapeutic opportunities. Int. J. Mol. Sci..

[50] Curzon P., Zhang M., Radek R.J., Fox G.B., Buccafusco J.J. (2009). Methods of Behavior Analysis in Neuroscience.

[51] Johnson K.R., Erway L.C., Cook S.A., Willott J.F., Zheng Q.Y. (1997). A major gene affecting age-related hearing loss in C57BL/6J mice. Hear. Res..

[52] Ison J.R., Allen P.D., O'Neill W.E. (2007). Age-related hearing loss in C57BL/6J mice has both frequency-specific and non-frequency-specific components that produce a hyperacusis-like exaggeration of the acoustic startle reflex. J. Assoc. Res. Otolaryngol..

[53] Cui H., Diedrich J.K., Wu D.C., Lim J.J., Nottingham R.M., Moresco J.J. (2023). Arg-tRNA synthetase links inflammatory metabolism to RNA splicing and nuclear trafficking via SRRM2. Nat. Cell Biol..

[54] Yannay-Cohen N., Carmi-Levy I., Kay G., Yang C.M., Han J.M., Kemeny D.M. (2009). LysRS serves as a key signaling molecule in the immune response by regulating gene expression. Mol. Cell.

[55] Han J., Back S.H., Hur J., Lin Y.H., Gildersleeve R., Shan J. (2013). ER-stress-induced transcriptional regulation increases protein synthesis leading to cell death. Nat. Cell Biol..

[56] Reiling J.H., Sabatini D.M. (2006). Stress and mTORture signaling. Oncogene.

[57] Parra-Peralbo E., Talamillo A., Barrio R. (2021). Origin and development of the adipose tissue, a key organ in physiology and disease. Front. Cell Dev. Biol..

[58] Frühbeck G. (2008). Overview of adipose tissue and its role in obesity and metabolic disorders. Methods Mol. Biol..

[59] Hawley J.A., Lessard S.J. (2008). Exercise training-induced improvements in insulin action. Acta Physiol. (Oxf).

[60] Yu D., Tomasiewicz J.L., Yang S.E., Miller B.R., Wakai M.H., Sherman D.S. (2019). Calorie-restriction-induced insulin sensitivity is mediated by adipose mTORC2 and not required for lifespan extension. Cell Rep..

[61] Guo D., Zhang A., Zou T., Ding R., Chen K., Pan Y. (2022). The influence of metabolic syndrome on age-related hearing loss from the perspective of mitochondrial dysfunction. Front. Aging Neurosci..

[62] Mittal R., McKenna K., Keith G., Lemos J.R.N., Mittal J., Hirani K. (2024). A systematic review of the association of Type I diabetes with sensorineural hearing loss. PLoS One.

[63] Deng Y., Chen S., Hu J. (2023). Diabetes mellitus and hearing loss. Mol. Med..

[64] Samocha-Bonet D., Wu B., Ryugo D.K. (2021). Diabetes mellitus and hearing loss: a review. Ageing Res. Rev..

[65] Buttgereit F., Brand M.D. (1995). A hierarchy of ATP-consuming processes in mammalian cells. Biochem. J..

[66] Jozefczuk J., Drews K., Adjaye J. (2012). Preparation of mouse embryonic fibroblast cells suitable for culturing human embryonic and induced pluripotent stem cells. J. Vis. Exp..

[67] Köhrer C., Rajbhandary U.L. (2008). The many applications of acid urea polyacrylamide gel electrophoresis to studies of tRNAs and aminoacyl-tRNA synthetases. Methods.

[68] Taicher G.Z., Tinsley F.C., Reiderman A., Heiman M.L. (2003). Quantitative magnetic resonance (QMR) method for bone and whole-body-composition analysis. Anal. Bioanal. Chem..

[69] Tinsley F.C., Taicher G.Z., Heiman M.L. (2004). Evaluation of a quantitative magnetic resonance method for mouse whole body composition analysis. Obes. Res..

[70] Bruss M.D., Khambatta C.F., Ruby M.A., Aggarwal I., Hellerstein M.K. (2010). Calorie restriction increases fatty acid synthesis and whole body fat oxidation rates. Am. J. Physiol. Endocrinol. Metab..

[71] Bray N.L., Pimentel H., Melsted P., Pachter L. (2016). Near-optimal probabilistic RNA-seq quantification. Nat. Biotechnol..

[72] Robinson M.D., McCarthy D.J., Smyth G.K. (2010). edgeR: a Bioconductor package for differential expression analysis of digital gene expression data. Bioinformatics.

[73] Ge S.X., Jung D., Yao R. (2020). ShinyGO: a graphical gene-set enrichment tool for animals and plants. Bioinformatics.

[74] Prospéro-García O., Huitrón-Resendiz S., Casalman S.C., Sánchez-Alavez M., Díaz-Ruiz O., Navarro L. (1999). Feline immunodeficiency virus envelope protein (FIVgp120) causes electrophysiological alterations in rats. Brain Res..

[75] Shvarev Y.N. (1994). Brainstem auditory evoked potentials characteristics in mice: the effect of genotype. Hear. Res..

[76] Drabkin M., Jean M.M., Noy Y., Halperin D., Yogev Y., Wormser O. (2024). SMARCA4 mutation causes human otosclerosis and a similar phenotype in mice. J. Med. Genet..

[77] Matsumura K., Kumar T.P., Guddanti T., Yan Y., Blackburn S.L., McBride D.W. (2019). Neurobehavioral deficits after subarachnoid hemorrhage in mice: sensitivity analysis and development of a new composite score. J. Am. Heart Assoc..

