# Supplementary material for: Murine nuclear tyrosyl-tRNA synthetase deficiency leads to fat storage deficiency and hearing loss
Source: J Biol Chem. 2024 Sep 12;300(10):107756. doi: 10.1016/j.jbc.2024.107756 (PMC11470617; doi:10.1016/j.jbc.2024.107756)
Supplement: Supporting Information [file mmc2.pdf]

Supporting Information

"Murine Nuclear Tyrosyl-tRNA Synthetase Deficiency Leads to Fat Storage Deficiency and Hearing Loss"

Julia A. Jones, Jiadong Zhou, Jianjie Dong, Salvador Huitron-Resendiz, Ely Boussaty, Eduardo Chavez, Na Wei, Calin Dan Dumitru, Yosuke Morodomi, Taisuke Kanaji, Allen F. Ryan, Rick Friedman, Tong Zhou, Sachiko Kanaji, Matthew Wortham, Simon Schenk, Amanda J. Roberts, and Xiang-Lei Yang

<u>This file includes:</u>

Figure S1: Generation of a nuclear TyrRS deficient mouse model and metabolic abnormalities of the *Yars*$^{\Delta NLS/\Delta NLS}$ MEFs.

Figure S2: *Yars*$^{\Delta NLS}$ mice have a normal lipid distribution and adipogenesis capacity and metabolically active organs are relatively proportional to *Yars*$^{+/+}$ mice.

Figure S3: Female *Yars*$^{\Delta NLS}$ mice display a similar insulin and glucose metabolism as the males, and there is no apparent insulin secretion defect.

Figure S4: Insulin signaling of metabolically active tissues after insulin stimulation reveals mild differences in *Yars*$^{\Delta NLS}$ mice and no difference in IGF-1.

Figure S5: Transcriptomic analysis uncovers transcriptional differences between *Yars*$^{\Delta NLS}$ white adipose tissue and liver.

Figure S6: The *Yars*$^{\Delta NLS}$ mice have a reduced startle response, and normal neuromuscular function.

Figure S7: The *Yars*$^{\Delta NLS}$ mice have a reduced peak amplitude and auditory threshold.

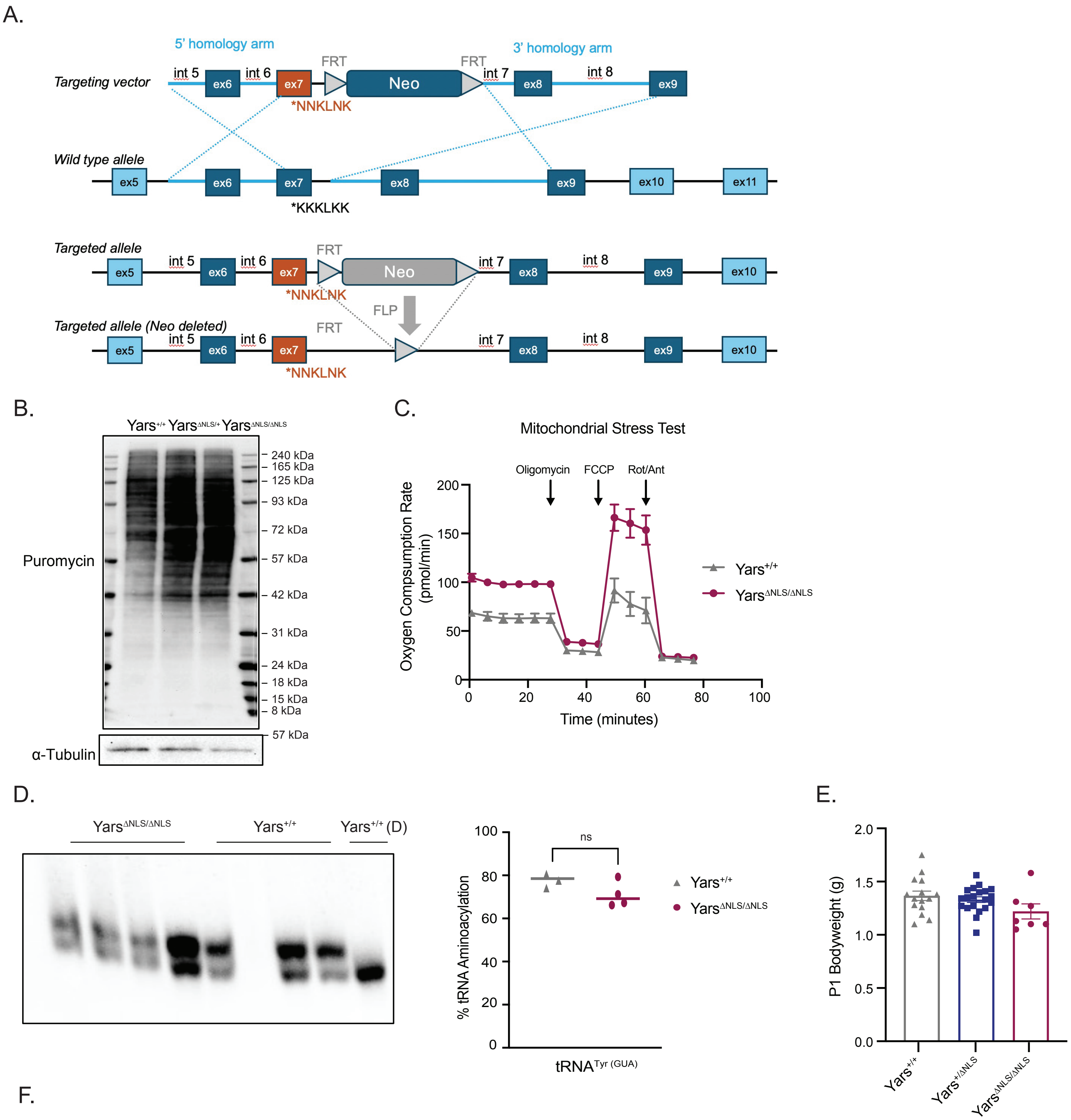

F.

Genotype	Male	Female
+/+	28 (29%)	31 (33%)
+/ΔNLS	48 (49%)	46 (49%)
ΔNLS/ΔNLS	21 (22%)	16 (17%)
Total	97 (51%)	93 (49%)

Figure S1.

Figure S1. Generation of a nuclear TyrRS deficient mouse model and metabolic abnormalities of the *Yars*$^{\Delta NLS/\Delta NLS}$ MEFs.

A. Targeting construct design and knock-in strategy for mutating the NLS from ^{242}KKKLKK^{247} to ^{242}NNKLNK^{247} in Exon 7 of the Yars1 gene. An FRT-flanked Neomycin cassette was introduced in the adjacent intron that was later bred out to create the ΔNLS-Neo-Out strain.
B. Western blot analysis of the puromycin incorporation assay (SUnSET) on MEFs.
C. Mitochondrial respiration stress test of MEFs using the Agilent Seahorse XFe96 Analyzer. Analysis indicated the following significant differences where "****"= $p<0.0001$ and "ns"=not significant using an Unpaired Welch's t-test: ATP production (****), basal respiration (****), proton leak (****), maximal respiration (****), spare respiratory capacity (****), non-mitochondrial O_2 consumption (ns), coupling efficiency (ns). FCCP: Carbonyl cyanide-4 (trifluoromethoxy) phenylhydrazone, Rot/Ant: rotenone and antimycin A
D. Acidic northern blot separating acylated and deacylated tRNA$^{Tyr\text{-}(GUA)}$ in liver and quantification of the charged species and uncharged species. (D): Deacetylated. Three to four per genotype. Unpaired Welch's t-test.
E. Bodyweight of newborn pups at day 1 (P1). One-way ANOVA, not significant.
F. Mice are born at approximate Mendelian ratio (25%, 50%, 25%). Chi-square analysis, not significant.

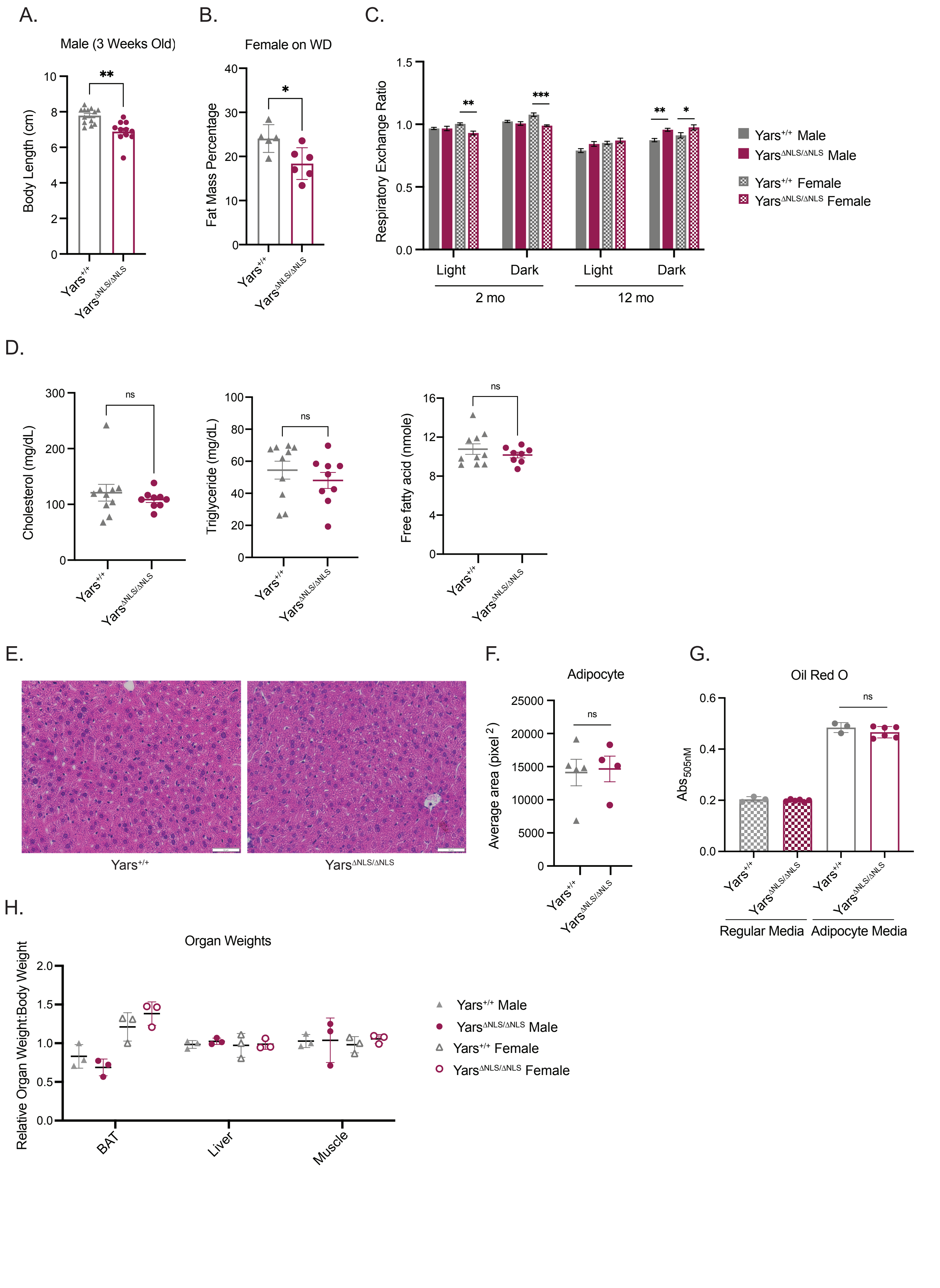
A.
Male (3 Weeks Old)
Body Length (cm)
**
Yars+/+
YarsΔNLS/ΔNLS
B.
Female on WD
Fat Mass Percentage
*
Yars+/+
YarsΔNLS/ΔNLS
C.
Respiratory Exchange Ratio
**
***
**
*
Light
Dark
Light
Dark
2 mo
12 mo
Yars+/+ Male
YarsΔNLS/ΔNLS Male
Yars+/+ Female
YarsΔNLS/ΔNLS Female
D.
Cholesterol (mg/dL)
ns
Triglyceride (mg/dL)
ns
Free fatty acid (nmole)
ns
Yars+/+
YarsΔNLS/ΔNLS
E.
Yars+/+
YarsΔNLS/ΔNLS
F.
Adipocyte
Average area (pixel 2)
ns
Yars+/+
YarsΔNLS/ΔNLS
G.
Oil Red O
Abs505nM
ns
Yars+/+
YarsΔNLS/ΔNLS
Regular Media
Adipocyte Media
H.
Organ Weights
Relative Organ Weight:Body Weight
BAT
Liver
Muscle
Yars+/+ Male
YarsΔNLS/ΔNLS Male
Yars+/+ Female
YarsΔNLS/ΔNLS Female

Figure S2.

Figure S2. *Yars*$^{\Delta NLS}$ mice have a normal lipid distribution and adipogenesis capacity and metabolically active organs are relatively proportional to *Yars*$^{+/+}$mice.

A. Male body length at 3 weeks old measured from tip of the nose to the base of the tail.
B. Five-month-old female mice fed Western Diet "WD" (Research Diets, Cat# D12079B) for 12 weeks had fat mass measured at the end point by EchoMRI. Unpaired Welch's t-test. *Yars*$^{+/+}$ N=5, *Yars*$^{\Delta NLS/\Delta NLS}$ N=6.
C. Average respiratory exchange ratio calculated from VO_2/VCO_2 at 2 and 12 months for both sexes over the light and dark cycles. 2-way ANOVA using Tukey's comparison analysis. Two-month sample size: Male: *Yars*$^{+/+}$ N=8, *Yars*$^{\Delta NLS/\Delta NLS}$ N=13; Female *Yars*$^{+/+}$ N=7, *Yars*$^{\Delta NLS/\Delta NLS}$ N=7; Twelve-month sample size: Male: *Yars*$^{+/+}$ N=4, *Yars*$^{\Delta NLS/\Delta NLS}$ N=4; Female *Yars*$^{+/+}$ N=4, *Yars*$^{\Delta NLS/\Delta NLS}$ N=4. Time in chamber: 72 hours for 2-month-old mice; 144 hours for 12-month-old.
D. Serum cholesterol, triglycerides, and free fatty acids (FFA) measured in 3-month-old male mice. Unpaired Welch's t-test. *Yars*$^{+/+}$ N=10, *Yars*$^{\Delta NLS/\Delta NLS}$ N=9 (except FFA, N=8).
E. Representative images of liver H&E-stained sections of 4-month-old male mice. Scale bar 50 µm. *Yars*$^{+/+}$ N=4, *Yars*$^{\Delta NLS/\Delta NLS}$ N=4
F. Epidydimal fat was processed for H&E and adipocytes per cross section were counted and sized using AdipoCount software (CSBIO). *Yars*$^{+/+}$ N=4, *Yars*$^{\Delta NLS/\Delta NLS}$ N=4. Unpaired Welch's t-test.
G. MEFs differentiated into adipocytes, stained with oil red-O, and results shown are quantified as total 505nm signal. 3-6 independent embryonic clones per genotype. Unpaired Welch's t-test on adipocyte media samples.
H. Tissues dissected from 6-month-old mice were weighed and normalized to body weight. *Yars*$^{+/+}$ N=3, *Yars*$^{\Delta NLS/\Delta NLS}$ N=3; BAT: interscapular brown adipose tissue, Muscle: hindlimb skeletal muscle.

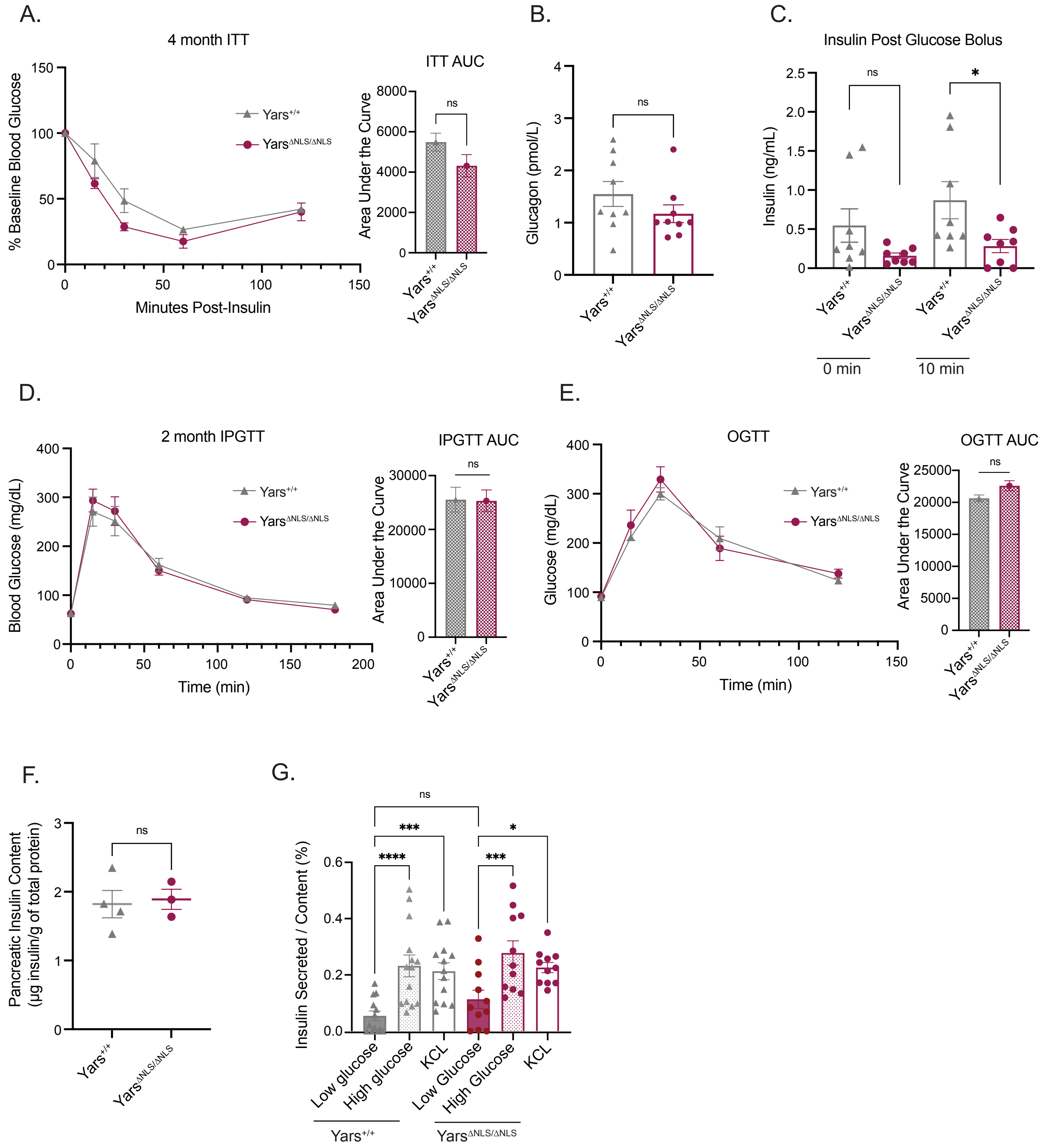

A.
4 month ITT
% Baseline Blood Glucose
Minutes Post-Insulin
Yars$^{+/+}$
Yars$^{\Delta NLS/\Delta NLS}$
ITT AUC
Area Under the Curve
ns
B.
Glucagon (pmol/L)
ns
C.
Insulin Post Glucose Bolus
Insulin (ng/mL)
ns
*
0 min
10 min
D.
2 month IPGTT
Blood Glucose (mg/dL)
Time (min)
IPGTT AUC
Area Under the Curve
ns
E.
OGTT
Glucose (mg/dL)
Time (min)
OGTT AUC
Area Under the Curve
ns
F.
Pancreatic Insulin Content (µg insulin/g of total protein)
ns
G.
Insulin Secreted / Content (%)
ns
***
****
*
***
Low glucose
High glucose
KCL
Low Glucose
High Glucose
KCL
Yars$^{+/+}$
Yars$^{\Delta NLS/\Delta NLS}$

Figure S3.

Figure S3. Female *Yars*$^{\Delta NLS}$ mice display a similar insulin and glucose metabolism as the males, and there is no apparent insulin secretion defect.

A. Insulin tolerance test (ITT) of 4-month-old female mice after fasting for 6 hours and injected intraperitoneally with insulin (1 U/kg). Analyzed by repeated measures two-way ANOVA with Sidak's analysis. Area under the curve (AUC) calculated and analyzed by unpaired Welch's t-test. *Yars*$^{+/+}$ N=4, *Yars*$^{\Delta NLS/\Delta NLS}$ N=4.
B. Plasma glucagon was measured in 4-month-old male mice after a 16 hour fast. Unpaired Welch's t-test. *Yars*$^{+/+}$ N=9, *Yars*$^{\Delta NLS/\Delta NLS}$ N=9.
C. Plasma insulin at 0- and 10-minutes post glucose bolus (2 g/kg) in 3–4-month-old female mice. Mice fasted 6 hours before injection. Repeated measures two-way ANOVA with Fisher's LSD test. *Yars*$^{+/+}$ N=8, *Yars*$^{\Delta NLS/\Delta NLS}$ N=8.
D. Blood glucose levels during an intraperitoneal glucose tolerance test (IPGTT) of 2-month-old female mice after fasting for 16 hours and injected with glucose (2 g/kg). Analyzed by repeated measures two-way ANOVA with Sidak's analysis. Area under the curve (AUC) calculated and analyzed by unpaired Welch's t-test. *Yars*$^{+/+}$ N=8, *Yars*$^{\Delta NLS/\Delta NLS}$ N=8.
E. Blood glucose levels during an oral glucose tolerance test (OGTT) of 4-5 month old male mice after fasting for 16 hours and oral gavaged with glucose (2 g/kg). Area under the curve (AUC) calculated and analyzed by unpaired Welch's t-test. *Yars*$^{+/+}$ N=10, *Yars*$^{\Delta NLS/\Delta NLS}$ N=8.
F. Total pancreatic insulin content was measured by ELISA and normalized to the total protein content by BCA analysis. Mice were fasted for 4 hours and pancreases were mechanically homogenized in 80% EtOH/HCl. Unpaired Welch's t-test.
G. The ratio of insulin secreted over the insulin content of individual islets under low glucose, high glucose, and KCl media. GSIS: Glucose-stimulated insulin secretion. Each datapoint represents 5 islets. Male mice were 3-4 months old at time of extraction. Two-way ANOVA with Tukey's comparison analysis.

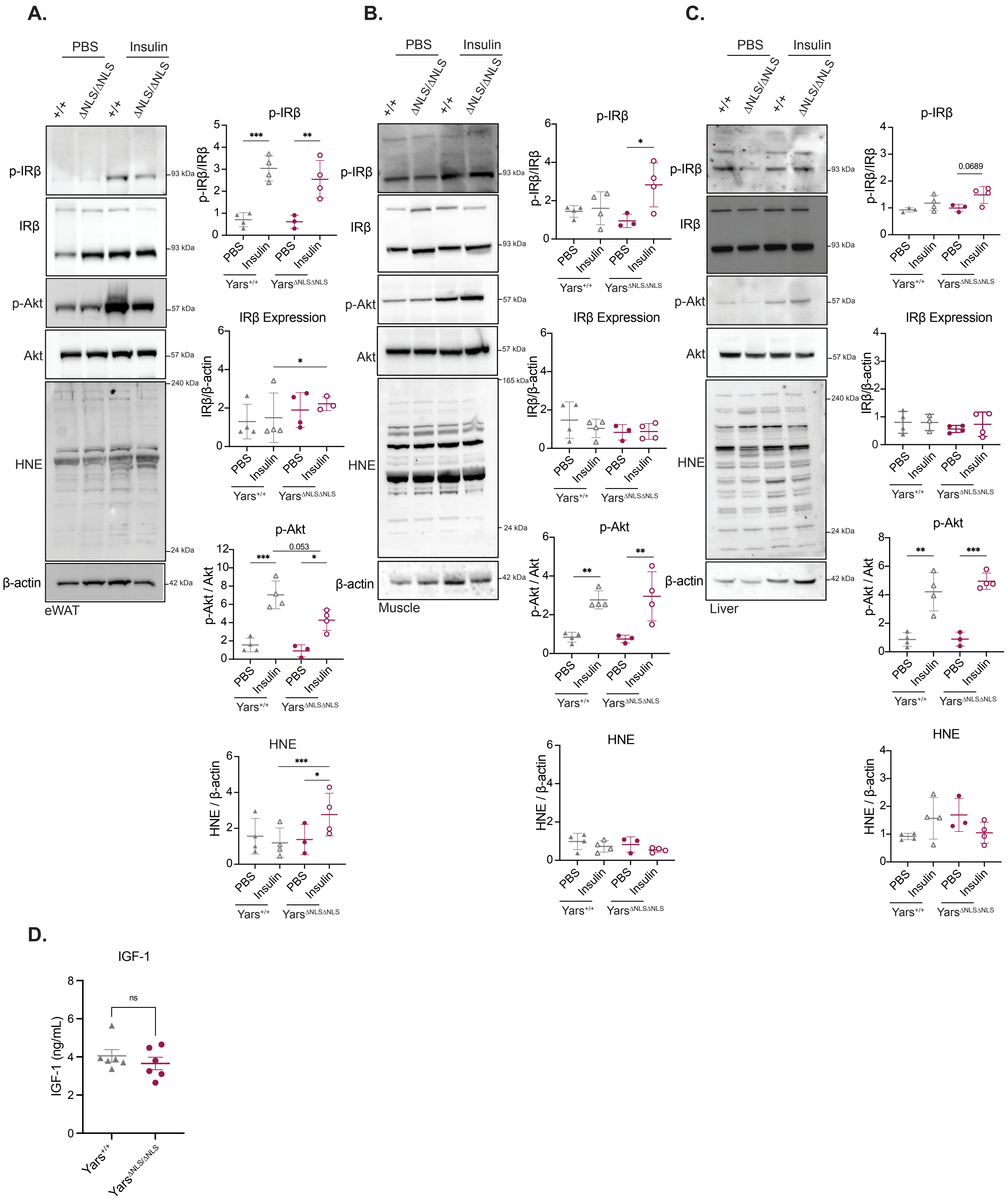
A.
B.
C.
D.
PBS
Insulin
+/+
ΔNLS/ΔNLS
p-IRβ
IRβ
p-Akt
Akt
HNE
β-actin
93 kDa
57 kDa
240 kDa
165 kDa
24 kDa
42 kDa
eWAT
Muscle
Liver
p-IRβ/IRβ
IRβ Expression
IRβ/β-actin
p-Akt / Akt
HNE / β-actin
0.053
0.0689
Yars+/+
YarsΔNLSΔNLS
IGF-1
IGF-1 (ng/mL)
ns

Figure S4.

Figure S4. Insulin signaling of metabolically active tissues after insulin stimulation reveals mild differences in *Yars*$^{\Delta NLS}$ mice and no difference in IGF-1. A-C: Male mice were injected with PBS or Insulin (1 U/kg), sacrificed after 15 min, and tissues extracted. 3-4 mice per group. Quantification of western blots where signal intensities were normalized to a wildtype PBS treated mouse and set to 1. Two-way ANOVA with Tukey's comparison analysis.

A. Representative western blot of epididymal white adipose tissue (eWAT) with or without insulin stimulation examining the noted targets of insulin signaling and 4-Hydroxynonenal (4-HNE). 20 μg loaded.
B. Representative western blot of skeletal hindlimb muscle with or without insulin stimulation examining the noted targets of insulin signaling and 4-Hydroxynonenal (4-HNE). 20 μg loaded.
C. Representative western blot of liver with or without insulin stimulation examining the noted targets of insulin signaling and 4-Hydroxynonenal (4-HNE). 10 μg loaded.
D. Serum insulin-like growth factor (IGF-1) in male mice 3-4 months old. Unpaired Welch's t-test. *Yars*$^{+/+}$ N=6, *Yars*$^{\Delta NLS/\Delta NLS}$ N=6.

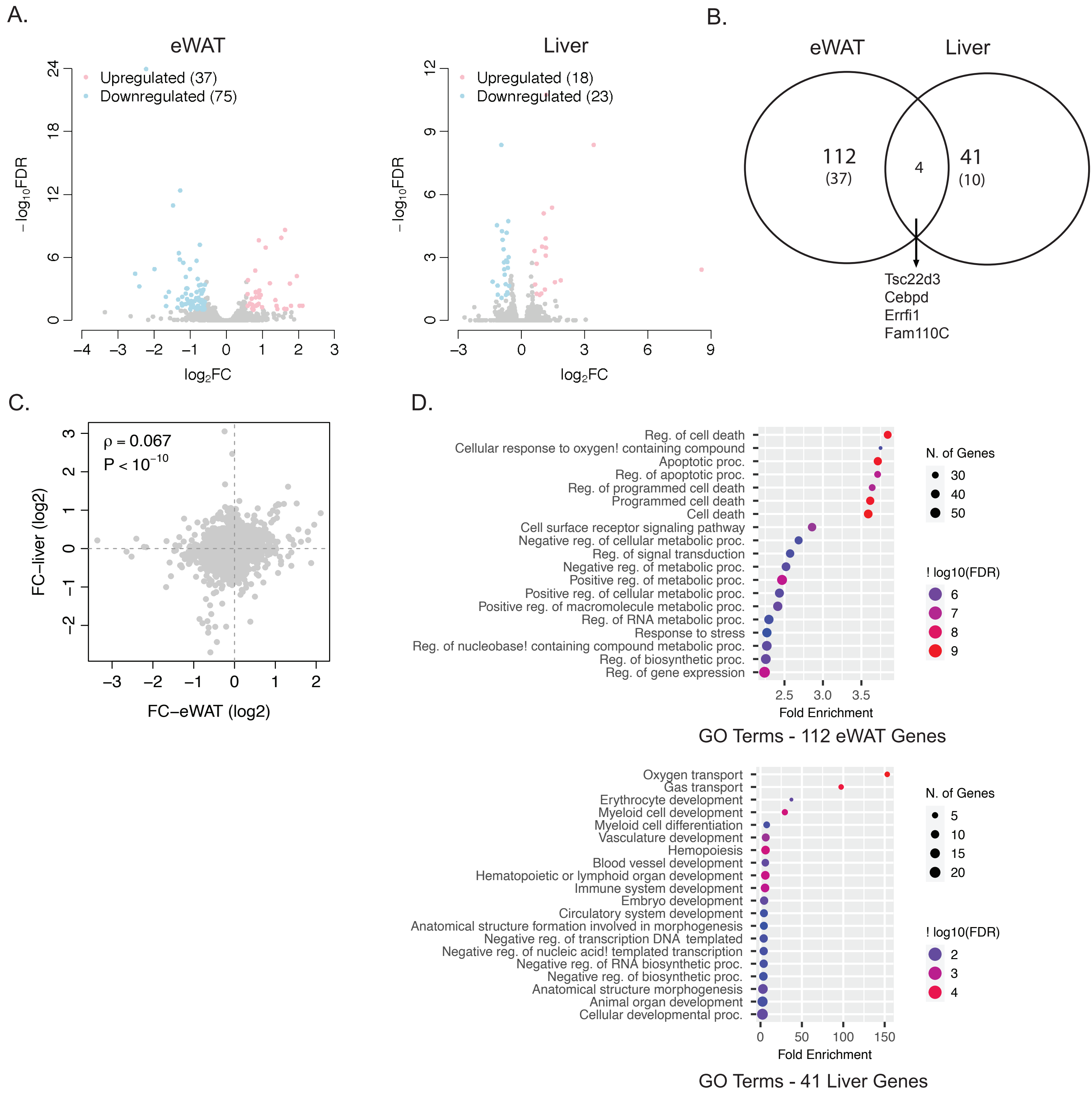

A.
eWAT
Upregulated (37)
Downregulated (75)
−log10FDR
log2FC
Liver
Upregulated (18)
Downregulated (23)
−log10FDR
log2FC
B.
eWAT
Liver
112
(37)
4
41
(10)
Tsc22d3
Cebpd
Errfi1
Fam110C
C.
ρ = 0.067
P < 10−10
FC−liver (log2)
FC−eWAT (log2)
D.
Reg. of cell death
Cellular response to oxygen! containing compound
Apoptotic proc.
Reg. of apoptotic proc.
Reg. of programmed cell death
Programmed cell death
Cell death
Cell surface receptor signaling pathway
Negative reg. of cellular metabolic proc.
Reg. of signal transduction
Negative reg. of metabolic proc.
Positive reg. of metabolic proc.
Positive reg. of cellular metabolic proc.
Positive reg. of macromolecule metabolic proc.
Reg. of RNA metabolic proc.
Response to stress
Reg. of nucleobase! containing compound metabolic proc.
Reg. of biosynthetic proc.
Reg. of gene expression
Fold Enrichment
N. of Genes
! log10(FDR)
GO Terms - 112 eWAT Genes
Oxygen transport
Gas transport
Erythrocyte development
Myeloid cell development
Myeloid cell differentiation
Vasculature development
Hemopoiesis
Blood vessel development
Hematopoietic or lymphoid organ development
Immune system development
Embryo development
Circulatory system development
Anatomical structure formation involved in morphogenesis
Negative reg. of transcription DNA templated
Negative reg. of nucleic acid! templated transcription
Negative reg. of RNA biosynthetic proc.
Negative reg. of biosynthetic proc.
Anatomical structure morphogenesis
Animal organ development
Cellular developmental proc.
Fold Enrichment
N. of Genes
! log10(FDR)
GO Terms - 41 Liver Genes

Figure S5.

Figure S5. Transcriptomic analysis uncovers transcriptional differences between *Yars*$^{\Delta NLS}$ white adipose tissue and liver. 12-week-old female mice were fasted for four hours prior to sacrifice and tissue harvesting followed by RNA extraction for bulk RNA-Sequencing.

A. Volcano plots of differentially expressed genes (DEG) of eWAT and liver between *Yars*$^{\Delta NLS}$ and wild type mice. Colored dots represent the up-regulated (pink) and down-regulated (blue) DEG with a false discovery rate (FDR) < 0.1 and a fold change (FC) > 1.5 as a cut-off threshold.
B. The FC correlation between the eWAT and liver gene expression changes. P: correlation coefficient ρ: p-value
C. Venn diagram of the significant eWAT versus liver DEG with the four overlapping genes listed. The number of known transcriptional regulator genes is listed in parentheses for each tissue (e.g., eWAT: 37/112 and liver: 10/41)
D. The Gene Ontology (GO) Biological Process terms associated with the eWAT DEG and liver DEG, respectively. Plots generated by ShinyGO 0.80. N.: Number; FDR: false discovery rate

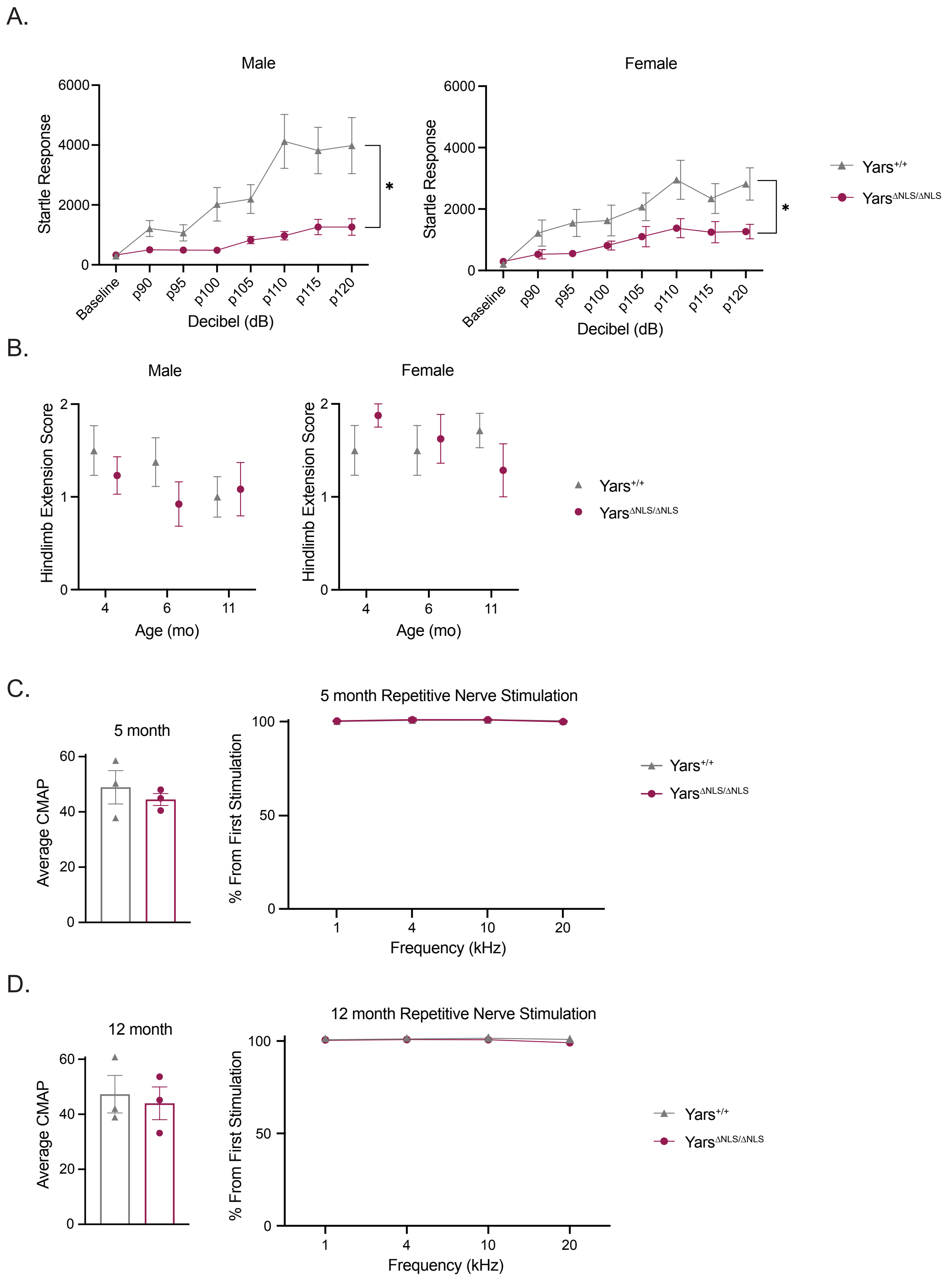
A.
Male
Female
Startle Response
6000
4000
2000
0
Baseline
p90
p95
p100
p105
p110
p115
p120
Decibel (dB)
*
Yars+/+
YarsΔNLS/ΔNLS
B.
Male
Female
Hindlimb Extension Score
2
1
0
4
6
11
Age (mo)
Yars+/+
YarsΔNLS/ΔNLS
C.
5 month
Average CMAP
60
40
20
0
5 month Repetitive Nerve Stimulation
% From First Stimulation
100
50
0
1
4
10
20
Frequency (kHz)
Yars+/+
YarsΔNLS/ΔNLS
D.
12 month
Average CMAP
60
40
20
0
12 month Repetitive Nerve Stimulation
% From First Stimulation
100
50
0
1
4
10
20
Frequency (kHz)
Yars+/+
YarsΔNLS/ΔNLS

Figure S6.

Figure S6. The *Yars*$^{\Delta NLS}$ mice have a reduced startle response, and normal neuromuscular function.

A. Acoustic startle reflex test using a brief tone stimulus between 90 and 120 dB. Two-way ANOVA. Males: *Yars*$^{+/+}$ N=9, *Yars*$^{\Delta NLS/\Delta NLS}$ N=13; Females: *Yars*$^{+/+}$ N=8, *Yars*$^{\Delta NLS/\Delta NLS}$ N=8
B. Quantification of the hindlimb extension reflex at 4, 6, and 11 months of age using a 0-2 scale where 2 is normal. Male: *Yars*$^{+/+}$ N=8, *Yars*$^{\Delta NLS/\Delta NLS}$ N=13; Female *Yars*$^{+/+}$ N=8, *Yars*$^{\Delta NLS/\Delta NLS}$ N=8.
C. Five-month male mice average compound muscle action potential (CMAP) and repetitive nerve stimulation (RNS) at the four indicated frequencies. *Yars*$^{+/+}$ N=3, *Yars*$^{\Delta NLS/\Delta NLS}$ N=3.
D. Twelve-month male mice average compound muscle action potential (CMAP) and repetitive nerve stimulation (RNS) at the four indicated frequencies. *Yars*$^{+/+}$ N=3, *Yars*$^{\Delta NLS/\Delta NLS}$ N=3

A.

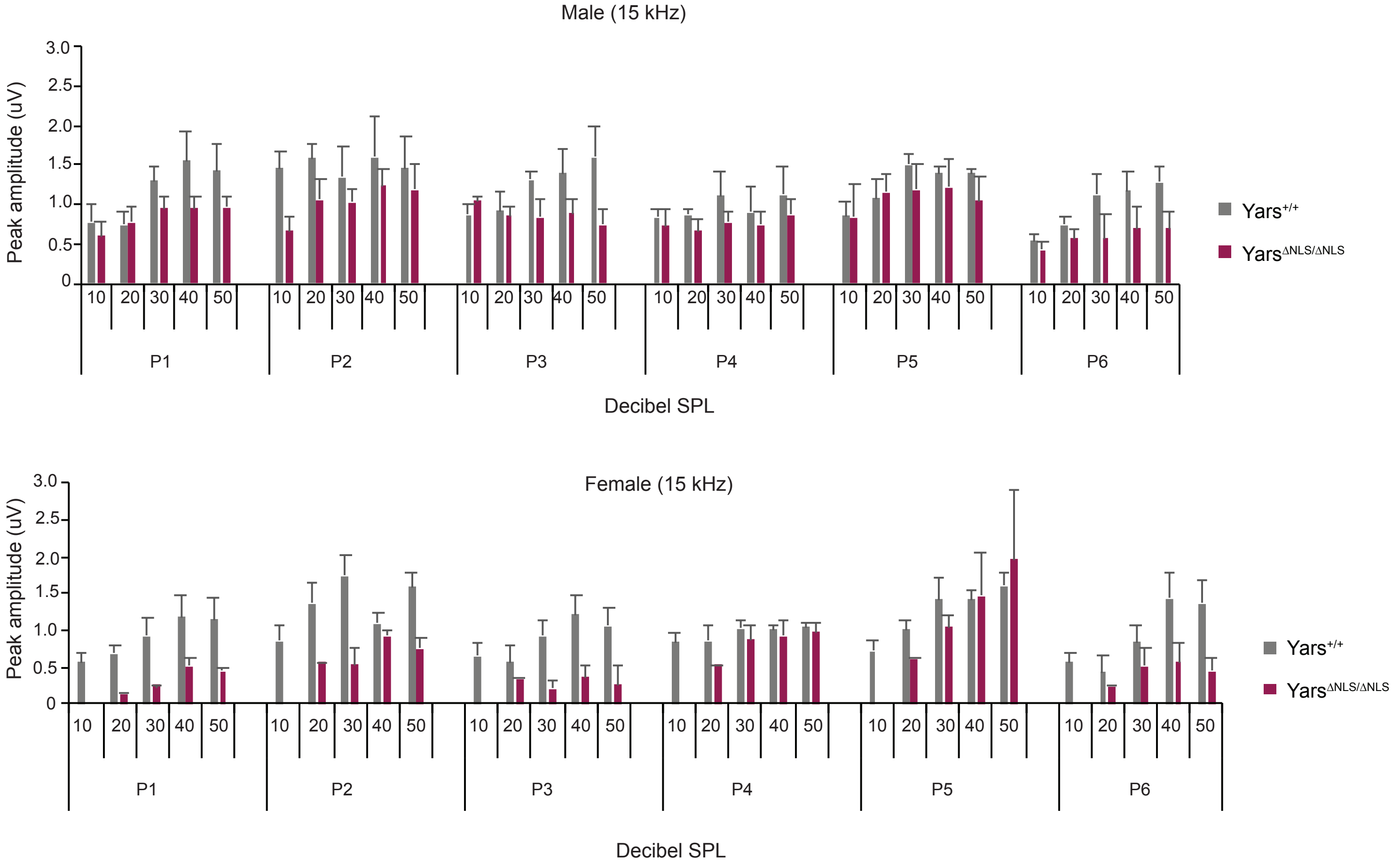
Male (15 kHz)
Peak amplitude (uV)
3.0
2.5
2.0
1.5
1.0
0.5
0
10 20 30 40 50
P1
P2
P3
P4
P5
P6
Decibel SPL
Yars+/+
YarsΔNLS/ΔNLS
Female (15 kHz)
Peak amplitude (uV)
Decibel SPL
Yars+/+
YarsΔNLS/ΔNLS

B.

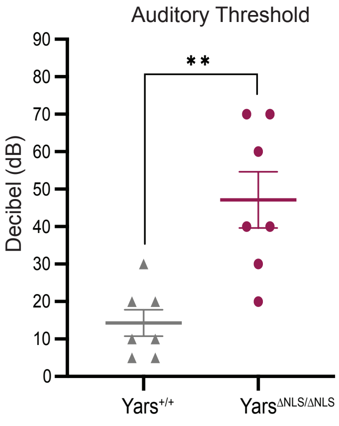
Auditory Threshold
**
Decibel (dB)
90
80
70
60
50
40
30
20
10
0
Yars+/+
YarsΔNLS/ΔNLS

Figure S7.

Figure S7. The *Yars*$^{\Delta NLS}$ mice have a reduced peak amplitude and auditory threshold.

A. Quantification of peak 1-6 amplitudes at 15 kHz from 0 - 50 dB of male and female mice at six months.

B. Auditory threshold at 4 kHz of male and female mice at six months. Unpaired Welch's t-test. *Yars*$^{+/+}$ N=7, *Yars*$^{\Delta NLS/\Delta NLS}$ N=7.
